# Association of Patient Belief About Success of Antibiotics for Appendicitis and Outcomes: A Secondary Analysis of the CODA Randomized Clinical Trial

**DOI:** 10.1001/jamasurg.2022.4765

**Published:** 2022-10-05

**Authors:** Writing Group for the CODA Collaborative, Irene Y. Zhang, Emily C. Voldal, Giana H. Davidson, Joshua M. Liao, Callie M. Thompson, Wesley H. Self, Lillian S. Kao, Jill Cherry-Bukowiec, Krishnan Raghavendran, Amy H. Kaji, Daniel A. DeUgarte, Eva Gonzalez, Katherine A. Mandell, Kristen Ohe, Nicole Siparsky, Thea P. Price, David C. Evans, Jesse Victory, William Chiang, Alan Jones, Matthew E. Kutcher, Hailie Ciomperlik, Mike K. Liang, Heather L. Evans, Brett A. Faine, Miriam Neufeld, Sabrina E. Sanchez, Anusha Krishnadasan, Bryan A. Comstock, Patrick J. Heagerty, Sarah O. Lawrence, Sarah E. Monsell, Erin E. C. Fannon, Larry G. Kessler, David A. Talan, David R. Flum

**Affiliations:** 1 Department of Surgery, Surgical Outcomes Research Center, University of Washington, Seattle; 2 Vanderbilt University Medical Center, Nashville, Tennessee; 3 Department of Surgery, University of Utah, Salt Lake City; 4 McGovern Medical School, The University of Texas Health Science Center at Houston, Houston; 5 Michigan Medicine, Ann Arbor; 6 Harbor–UCLA Medical Center, West Carson, California; 7 The Swedish Medical Center, Seattle, Washington; 8 Rush University Medical Center, Chicago, Illinois; 9 The Ohio State University Wexner Medical Center, Columbus; 10 Bellevue Hospital Center, NYU School of Medicine, New York, New York; 11 Tisch Hospital, NYU Langone Medical Center, New York, New York; 12 The University of Mississippi Medical Center, Jackson; 13 Lyndon B. Johnson General Hospital, University of Texas, Houston; 14 HCA Healthcare, University of Houston, Kingwood, Kingwood, Texas; 15 Harborview Medical Center, UW Medicine, Seattle, Washington; 16 The Medical University of South Carolina, Charleston; 17 University of Iowa Hospitals and Clinics, Iowa City; 18 Boston University Medical Center, Boston, Massachusetts; 19 Olive View–UCLA Medical Center, Los Angeles, California; 20 Ronald Reagan UCLA Medical Center, Los Angeles, California

## Abstract

**Importance:**

A patient’s belief in the likely success of a treatment may influence outcomes, but this has been understudied in surgical trials.

**Objective:**

To examine the association between patients’ baseline beliefs about the likelihood of treatment success with outcomes of antibiotics for appendicitis in the Comparison of Outcomes of Antibiotic Drugs and Appendectomy (CODA) trial.

**Design, Setting, and Participants:**

This was a secondary analysis of the CODA randomized clinical trial. Participants from 25 US medical centers were enrolled between May 3, 2016, and February 5, 2020. Included in the analysis were participants with appendicitis who were randomly assigned to receive antibiotics in the CODA trial. After informed consent but before randomization, participants who were assigned to receive antibiotics responded to a baseline survey including a question about how successful they believed antibiotics could be in treating their appendicitis.

**Interventions:**

Participants were categorized based on baseline survey responses into 1 of 3 belief groups: unsuccessful/unsure, intermediate, and completely successful.

**Main Outcomes and Measures:**

Three outcomes were assigned at 30 days: (1) appendectomy, (2) high decisional regret or dissatisfaction with treatment, and (3) persistent signs and symptoms (abdominal pain, tenderness, fever, or chills). Outcomes were compared across groups using adjusted risk differences (aRDs), with propensity score adjustment for sociodemographic and clinical factors.

**Results:**

Of the 776 study participants who were assigned antibiotic treatment in CODA, a total of 425 (mean [SD] age, 38.5 [13.6] years; 277 male [65%]) completed the baseline belief survey before knowing their treatment assignment. Baseline beliefs were as follows: 22% of participants (92 of 415) had an unsuccessful/unsure response, 51% (212 of 415) had an intermediate response, and 27% (111 of 415) had a completely successful response. Compared with the unsuccessful/unsure group, those who believed antibiotics could be completely successful had a 13–percentage point lower risk of appendectomy (aRD, −13.49; 95% CI, −24.57 to −2.40). The aRD between those with intermediate vs unsuccessful/unsure beliefs was −5.68 (95% CI, −16.57 to 5.20). Compared with the unsuccessful/unsure group, those with intermediate beliefs had a lower risk of persistent signs and symptoms (aRD, −15.72; 95% CI, −29.71 to −1.72), with directionally similar results for the completely successful group (aRD, −15.14; 95% CI, −30.56 to 0.28).

**Conclusions and Relevance:**

Positive patient beliefs about the likely success of antibiotics for appendicitis were associated with a lower risk of appendectomy and with resolution of signs and symptoms by 30 days. Pathways relating beliefs to outcomes and the potential modifiability of beliefs to improve outcomes merit further investigation.

**Trial Registration:**

ClinicalTrials.gov Identifier: NCT02800785

## Key Points

**Question:**

How are patients’ beliefs about treatment success associated with outcomes when using antibiotics for the treatment of appendicitis?

**Findings:**

The secondary analysis of the Comparison of Outcomes of Antibiotic Drugs and Appendectomy (CODA) randomized clinical trial of 425 participants found that positive beliefs at baseline about the success of antibiotics for appendicitis were associated with a substantially lower risk of appendectomy after antibiotics and with resolution of signs and symptoms by 30 days.

**Meaning:**

Understanding patients’ beliefs about treatment success when making decisions about treatment for appendicitis may be important; pathways relating beliefs to outcomes and the potential modifiability of beliefs to improve outcomes merit further investigation.

## Teaser

This secondary analysis of the Comparison of Outcomes of Antibiotic Drugs and Appendectomy (CODA) randomized clinical trial investigates the association between patients’ belief in treatment success and outcomes regarding antibiotic management for appendicitis.

## Introduction

A patient’s belief in the likelihood of success of a treatment can influence both objectively measured outcomes and the perception of success. Best demonstrated in studies of placebos, the effect has been reproducibly identified in studies of sham interventions compared with surgical procedures, medications, and other therapies.^1,2,3,4^ There may be a more general phenomenon in which a patient’s belief in success with specific treatments—not just placebos—might influence outcomes. However, clinical trials rarely measure patients’ beliefs about success regarding the treatments being offered, and the potential effect of beliefs in this context is not well understood.

The recently completed Comparison of Outcomes of Antibiotic Drugs and Appendectomy (CODA) trial was a pragmatic, nonblinded, noninferiority, randomized clinical trial of appendectomy vs antibiotics for acute appendicitis performed at 25 medical centers across the US, funded by the Patient-Centered Outcomes Research Institute (PCORI).^5^ It included 1552 participants who were randomly assigned to either antibiotics or appendectomy. The primary analysis found that antibiotics were noninferior to surgical procedure for general health status at 30 days.^6^ Subsequent secondary analyses have investigated the clinical factors of participants who were randomly assigned to receive antibiotics but underwent appendectomy within 30 days of randomization. These analyses have identified appendicolith and appendiceal diameter as potentially important clinical factors; notably, the analyses have also shown that there remains substantial variation in outcome not explained by these and other clinical factors.^7^

In the CODA trial, participants were asked at enrollment about their beliefs concerning treatment success with antibiotics. We conducted a secondary analysis among a subset of CODA participants who were randomly assigned to receive antibiotics for appendicitis and who completed this survey before they became aware of their randomization assignment, exploring the association between patient beliefs and outcomes.

## Methods

### Study Cohort

We analyzed a subset of participants with appendicitis who were randomly assigned to receive antibiotics in the CODA trial. Details of the CODA trial design have been previously published (Supplements 1 and 2 and eAppendix 5 in Supplement 3).^5,6^ Institutional review boards at 25 clinical sites participating in the University of Washington–based Comparative Effectiveness Research Translation Network (CERTAIN) approved the protocol, and the participants provided written informed consent. Participants from the following race and ethnicity groups were included: Asian, Black or African American, Hispanic, non-Hispanic White, and other (including American Indian or Alaska Native and Native Hawaiian or Other Pacific Islander) or multiracial. Participants were given a baseline survey containing a question about beliefs regarding antibiotics after viewing the informed consent materials and consenting to randomization. Only those individuals who were not aware of their treatment assignment at the time of the baseline survey were included in this analysis. This stipulation was made with the intent to analyze preexisting beliefs of patients rather than the potential dynamic of psychologically investing in the treatment they had been assigned. Participants were excluded from the analysis if they were already aware of their treatment assignment when answering the baseline survey or if the timing of their survey responses relative to the treatment assignment was not recorded. This study followed the Consolidated Standards of Reporting Trials (CONSORT) reporting guidelines.

### Belief in Success of Antibiotics

As part of the baseline survey, participants were asked, “Please indicate how successful you believe [antibiotics] could be in treating your appendicitis by choosing a number on a scale of 0 to 10, with 0 being unsuccessful, 5 being unsure, and 10 being a completely successful treatment of appendicitis.” Based on the distribution of responses, we categorized participants into belief groups: unsuccessful/unsure (0-5), intermediate (6-9), or completely successful (10). Divisions between groups were based on the divisions in the scale provided to participants, ie, 0, 5, and 10. Decisions about in which group the boundary points were included were based on a desire to group subjectively similar responses and to ensure that no group was too small for inference.

All participants answering this question had been given a standardized set of materials describing the 2 treatment groups, including either a pamphlet or a video explaining the outcomes and risks seen in previous studies of treating appendicitis with antibiotics or appendectomy. These materials contained statements like “1 of 10 patients experience a minor complication after surgery” and “3 of 4 antibiotics patients did not get appendicitis again.”^8^

### Additional Baseline Variables

Other patient factors, including demographic, clinical, and radiologic variables, were gathered at baseline and used to describe belief groups. These variables have been previously described^6,7,9^ and are summarized in eAppendix 1 in Supplement 3.

### Outcomes

This analysis focused on 3 binary outcomes within the first 30 days after randomization, a window commonly used to describe outcomes for the index episode of appendicitis and consistent with other secondary CODA analyses.^7,9^ Antibiotic treatment consisted of a 10-day course of oral and/or intravenous antibiotics. Appendectomy was recommended for participants in the antibiotics group if diffuse peritonitis or septic shock developed at any time or if worsening signs and symptoms developed after 48 hours of antibiotics; however, these criteria were not required to be met. In the absence of these conditions, participants were encouraged to continue taking antibiotics, and the decision to perform appendectomy was ultimately made by the treating clinician. Appendectomy status 30 days after randomization was determined by patient surveys.^7,9^

At 30 days after randomization, participants were surveyed about persistent signs and symptoms of appendicitis. Participants were asked whether they had experienced any of the following symptoms since their last follow-up with study coordinators: pain in the lower right abdominal area (yes/no), tenderness when the lower right abdomen is pressed (yes/no), and fever or body temperature greater than 101 °F (38.3 °C) or shaking chills (yes/no/unsure). Participants who responded yes to any of these questions were considered to have persistent signs and symptoms. Otherwise, participants answering no or unsure were considered to have resolution of signs and symptoms by 30 days. For participants who had responded to the 2-week survey, relevant ongoing symptoms could have occurred between 2 weeks and 4 weeks after randomization.

To assess participants’ feelings about their appendicitis treatment, we used a composite outcome of their reported decisional regret or dissatisfaction with treatment from the 30-day survey. To assess decisional regret regarding their appendicitis treatment, we used the validated Decisional Regret Scale^10^ asking participants to “Think about the decision to follow through with your initial treatment of appendectomy or antibiotics for your appendicitis. Please tell us how much you agree with the following statements.” Participants could respond strongly agree, agree, neither agree nor disagree, disagree, or strongly disagree to the following 5 statements: “It was the right decision; I regret the choice that was made; I would go for the same choice if I had to do it over again; the choice did me a lot of harm; and the decision was a wise one.” The Decisional Regret Scale ranges from 0 (no regret) to 100 (high regret), which we dichotomized as high decisional regret (>50) or not high decisional regret (≤50), which was consistent with prior CODA secondary analyses.^9^ Additionally, participants were asked, “How satisfied are you with your treatment for appendicitis?” with the following response options: very dissatisfied, somewhat dissatisfied, somewhat satisfied, or very satisfied. This was also dichotomized into dissatisfaction (very dissatisfied or somewhat dissatisfied) or no dissatisfaction (other responses), which was consistent with prior CODA secondary analyses.^9^ Previous analyses of the CODA data have shown that both dissatisfaction and high regret are uncommon and somewhat correlated^9^; to facilitate comparisons across belief groups with smaller sample sizes in this analysis, we defined a composite outcome for either high decisional regret or dissatisfaction at 30 days.

### Statistical Analysis

Baseline characteristics of the participants overall and by belief group were described using means (SDs) for continuous measures and number (percentage) for categorical variables. We described binary outcomes in each belief group using percentages and CIs, calculated using a log-binomial framework. In these and the methods described, missing data were imputed using multivariate imputation by chained equations (MICE; eAppendix 2 in Supplement 3) algorithms,^11^ and estimates were pooled using Rubin rules.^12^ Individuals who did not answer the survey question about beliefs but did respond to other parts of the baseline survey were included in the primary analyses using MICE to account for missing information about beliefs to reduce potential nonresponse bias.

Formal comparisons between belief groups were made with standardized risk differences (RDs) and 95% CIs, calculated by marginalizing logistic regression models.^13^ To account for potential confounding, adjusted risk differences (aRDs) were calculated using propensity score adjustment (eAppendix 3 in Supplement 3) for the following baseline factors: age, sex, body mass index, race, Hispanic ethnicity, health literacy help, education, low income or Medicaid/state program, average pain in the previous 7 days, appendiceal diameter, and appendicolith.

The main analysis examined appendectomy after antibiotics within the first 30 days of randomization as a binary outcome. As a secondary analysis, we created a Kaplan-Meier curve, stratified by belief group. We calculated hazard ratios (HRs) using Cox proportional hazard models within 2 separate time windows: 0 to 48 hours and 48 hours to 30 days. Analyses were performed in R statistical software, version 4.0.5 (R Project for Statistical Computing).

## Results

Among the 776 antibiotic-assigned participants in CODA, only 425 (mean [SD] age, 38.5 [13.6] years; 277 male [65%]; 148 female [35%]) who were not aware of their treatment assignment at the time of the baseline survey were included in this analysis. Participants identified with the following race and ethnicities: 23 Asian (5%), 31 Black or African American (7%), 191 Hispanic (45%), 234 non-Hispanic (55%); 261 White (62%), and 106 other/multiracial (25%) (Table). Among the included participants, 415 answered the question about beliefs, and an additional 10 did not answer this question but did respond to other parts of the baseline survey. Of the 351 participants who were excluded from this analysis, 256 were excluded because they were already aware of their treatment assignment when answering the baseline survey, and 95 were excluded because the timing of their survey responses relative to the treatment assignment was not recorded. Sociodemographic characteristics (Table) were generally similar to those in the complete antibiotics group in the CODA trial.^6^ Among the cohort, baseline beliefs about antibiotics were distributed as follows: 22% of participants (92 of 415) had an unsuccessful/unsure response, 51% (212 of 415) had an intermediate response, and 27% (111 of 415) had a completely successful response (eFigure in Supplement 3). Within the unsuccessful/unsure group, 97% of participants (89 of 92) scored their belief as a 5, which was identified on the scale as unsure. The Table shows the distribution of sociodemographic and clinical factors across belief groups, including the factors accounted for in the adjusted analyses.

**Table.** Sociodemographic and Clinical Characteristics of Participants Randomized to Antibiotics and Included in This Analysis, Both Overall and by Belief About the Success of Antibiotics^a^

Baseline characteristic	No. (%)			
	Overall^**b**^	Unsuccessful/unsure	Intermediate	Completely successful
No.	425	92	212	111
Age, mean (SD), y	38.48 (13.60)	39.52 (14.14)	37.36 (13.14)	39.85 (13.63)
Sex				
Male	277 (65)	58 (63)	140 (66)	71 (64)
Female	148 (35)	34 (37)	72 (34)	40 (36)
Race				
Asian	23 (5)	8 (9)	11 (5)	4 (4)
Black or African American	31 (7)	17 (19)	7 (3)	7 (6)
White	261 (62)	43 (47)	150 (71)	62 (56)
Other/multiracial^c^	106 (25)	23 (25)	42 (20)	38 (34)
Hispanic				
Yes	191 (45)	37 (40)	82 (39)	68 (61)
No	234 (55)	55 (60)	130 (61)	43 (39)
Education				
High school/GED or less	172 (41)	39 (42)	66 (31)	65 (59)
Some beyond high school/GED	249 (59)	53 (58)	145 (69)	46 (41)
Health literacy help				
Sometimes or more	65 (16)	13 (15)	24 (11)	27 (25)
Never or rarely	345 (84)	75 (85)	186 (89)	81 (75)
Low income or Medicaid/state program				
Yes	149 (45)	31 (42)	66 (39)	49 (58)
No	183 (55)	42 (58)	103 (61)	36 (42)
Charlson, mean (SD)	0.3 (0.6)	0.3 (0.7)	0.3 (0.6)	0.3 (0.5)
Body mass index, mean (SD)^d^	29.2 (7.0)	29.7 (8.1)	28.9 (6.6)	29.5 (6.8)
Alvarado, mean (SD)	6.5 (1.7)	6.5 (1.6)	6.5 (1.6)	6.5 (1.8)
White blood cell count, mean (SD), thousand/μL	13.0 (4.3)	13.2 (4.6)	13.0 (4.2)	12.9 (4.3)
Duration of symptoms				
<1 d	112 (26)	22 (24)	65 (31)	22 (20)
≥1 d	312 (74)	70 (76)	146 (69)	89 (80)
Average pain in the previous 7 d, mean (SD)	5.6 (3.0)	5.8 (2.9)	5.3 (2.9)	6.1 (3.1)
Fever				
Yes	113 (27)	26 (28)	54 (25)	31 (28)
No	312 (73)	66 (72)	158 (75)	80 (72)
Appendiceal diameter, mean (SD), mm	11.4 (2.8)	11.7 (2.8)	11.2 (2.9)	11.6 (2.7)
Appendicolith				
Yes	118 (28)	34 (37)	54 (25)	27 (24)
No	307 (72)	58 (63)	158 (75)	84 (76)

Abbreviation: GED, General Educational Development test.

SI conversion factor: To convert white blood cell count from thousand per microliter to × 10^9^ per liter, multiply by 0.001.

^a^ The following baseline variables were missing for more than 10% of patients: body mass index, missing in 133 of 425 participants; low income or Medicaid/state program missing in 93 of 425 participants; appendiceal diameter, missing in 56 of 425 participants (eAppendix 1 in Supplement 3).

^b^ Ten participants were missing data on belief and are included in the first column only.

^c^ Other race and ethnicity included American Indian or Alaska Native and Native Hawaiian or Other Pacific Islander.

^d^ Calculated as weight in kilograms divided by height in meters squared.

**Figure 1.**
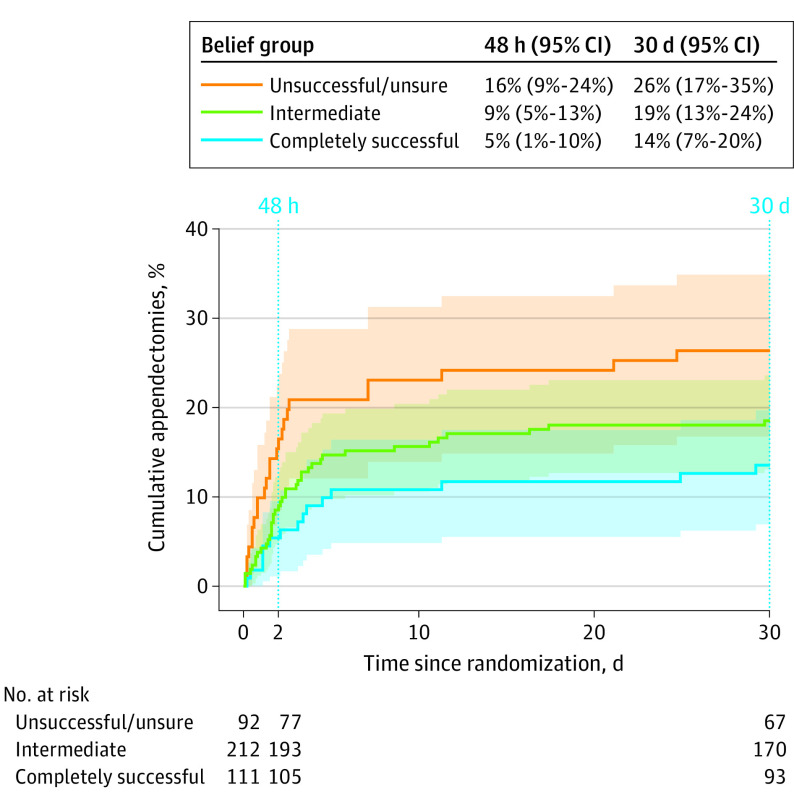
Cumulative Incidence of Appendectomy After Being Randomly Assigned to Antibiotics, by Belief Group Shaded regions correspond to 95% CIs. Cumulative incidence (95% CI) and number of participants still at risk for an appendectomy are shown at 48 hours and 30 days after randomization for each belief group. Ten participants were excluded from this figure for missing data on belief.

The cumulative incidence of appendectomy after antibiotics appeared to vary over time across belief groups but with wide CIs (Figure 1). Compared with the unsuccessful/unsure group, in the first 48 hours after randomization, those with intermediate beliefs had an HR for appendectomy of 0.49 (95% CI, 0.24-0.97), whereas those who believed antibiotics could be completely successful had an HR of 0.29 (95% CI, 0.11-0.77). Among those who did not have an appendectomy in the first 48 hours, the HR for 48 hours to 30 days was 0.88 (95% CI, 0.39-1.99) for intermediate vs unsuccessful/unsure and 0.71 (95% CI, 0.27-1.86) for completely successful vs unsuccessful/unsure.

**Figure 2.**
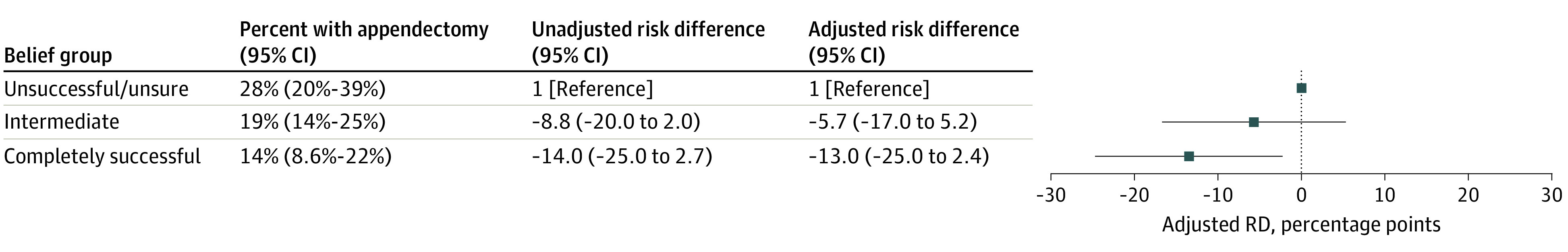
Association Between Belief and Appendectomy Within 30 Days With 95% CIs Estimates are pooled from multiply imputed data sets. Baseline factors accounted for in the adjusted risk differences (RDs) were age, sex, body mass index, race, Hispanic ethnicity, health literacy help, education, low income or Medicaid/state program, average pain in the previous 7 days, appendiceal diameter, and appendicolith. Information on appendectomy status at 30 days was missing for 14 participants (eAppendix 4 in Supplement 3 for counts from complete case data).

Our main analysis found that beliefs were associated with appendectomy after antibiotics within the first 30 days, based on both adjusted and unadjusted RDs (Figure 2). Overall, 20% (95% CI, 16%-24%) of the 425 participants had an appendectomy (based on full sample including imputed data; eAppendix 4 in Supplement 3 for complete case counts and percentages). Those who believed that antibiotics could be unsuccessful/unsure had the highest risk of appendectomy after antibiotics at 28% (95% CI, 20%-39%). The intermediate belief group had a lower risk of appendectomy at 19% (95% CI, 14%-25%), but the CI for the RD between the intermediate and unsuccessful/unsure groups included 0 (RD, −8.76; 95% CI, −19.53 to 2.01). Those who believed antibiotics could be completely successful had approximately half of the risk of appendectomy after antibiotics at 14% (95% CI, 8.6%-22%) compared with the unsuccessful/unsure group, with an RD of −14.08 percentage points (95% CI, −25.46 to −2.71). These associations generally persisted after adjusting for baseline sociodemographic and clinical factors. Compared with the unsuccessful/unsure group and after adjustment, those who believed that antibiotics could be completely successful had a 13–percentage point lower risk of appendectomy (aRD, −13.49; 95% CI, −24.57 to −2.40) (Figure 2). The aRD between those with intermediate vs unsuccessful/unsure beliefs was −5.68 (95% CI, −16.57 to 5.20).

**Figure 3.**
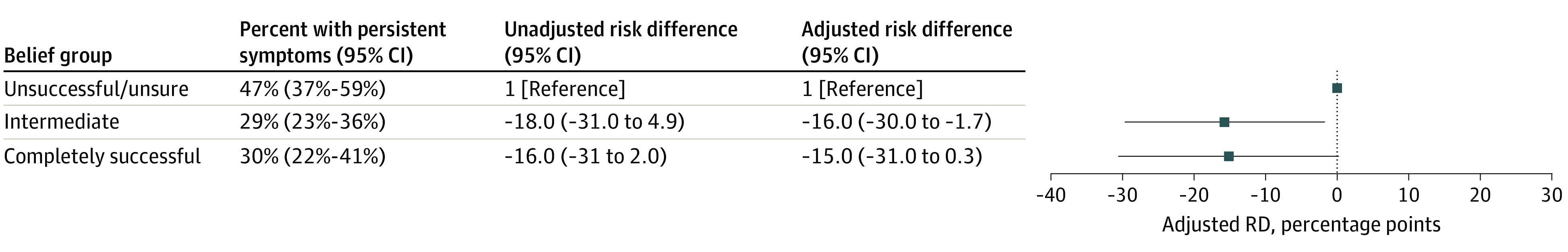
Association Between Belief and Signs or Symptoms of Appendicitis Up to 30 Days With 95% CIs Estimates are pooled from multiply imputed data sets. Baseline factors accounted for in the adjusted risk differences (RDs) were age, sex, body mass index, race, Hispanic ethnicity, health literacy help, education, low income or Medicaid/state program, average pain in the previous 7 days, appendiceal diameter, and appendicolith. Information on persistent signs and symptoms at 30 days was missing for 41 participants (eAppendix 4 in Supplement 3 for counts from complete case data).

Beliefs were also associated with patient-reported persistent signs and symptoms (abdominal pain, tenderness, fever, or chills) up to 30 days (Figure 3). Overall, 33% (95% CI, 29%-38%) of participants reported persistent signs and symptoms: 47% (95% CI, 37%-59%) in the unsuccessful/unsure belief group, 29% (95% CI, 23%-36%) in the intermediate belief group, and 30% (95% CI, 22%-41%) in the completely successful belief group. In the unadjusted analysis, both the intermediate and completely successful belief groups had a lower risk of persistent signs and symptoms compared with the unsuccessful/unsure group (RD, −18.02; 95% CI, −31.13 to −4.90 for intermediate vs unsuccessful/unsure; RD, −16.47; 95% CI, −30.90 to −2.04 for completely successful vs unsuccessful/unsure). Adjusted analyses also found that those with intermediate beliefs, compared with the unsuccessful/unsure group, had a lower risk of persistent signs or symptoms by −15.72 percentage points (95% CI, −29.71 to −1.72). Directionally similar results were noted for those who believed antibiotics could be completely successful, but after adjustment, the CI included 0 (aRD, −15.14; 95% CI, −30.56 to 0.28).

**Figure 4.**
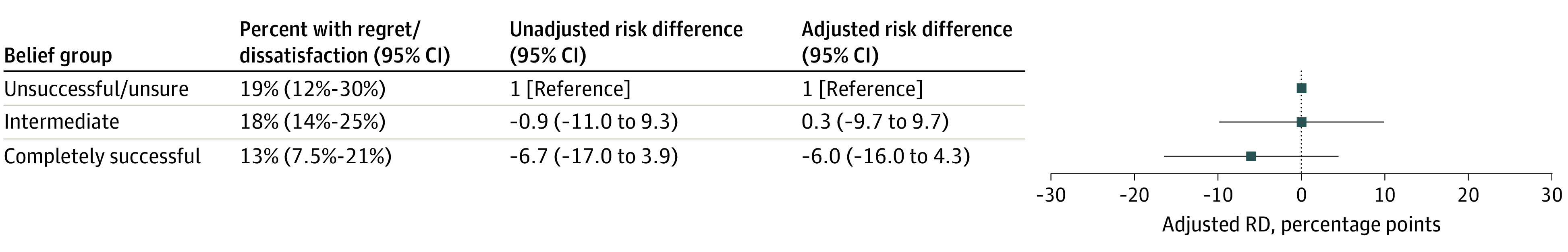
Association Between Belief and High Regret or Dissatisfaction at 30 Days With 95% CIs Estimates are pooled from multiply imputed data sets. Baseline factors accounted for in the adjusted risk differences (RDs) were age, sex, body mass index, race, Hispanic ethnicity, health literacy help, education, low income or Medicaid/state program, average pain in the previous 7 days, appendiceal diameter, and appendicolith. Information on regret or dissatisfaction at 30 days was missing for 50 participants (eAppendix 4 in Supplement 3 for counts from complete case data).

Overall, 17% (95% CI, 14%-21%) of participants reported high decisional regret or dissatisfaction with treatment at 30 days. High decisional regret or dissatisfaction was reported by 19% (95% CI, 12%-30%) of the unsuccessful/unsure belief group, 18% (95% CI, 14%-25%) of the intermediate belief group, and 13% (95% CI, 8%-21%) of the completely successful belief group (Figure 4). Beliefs were not found to be associated with high decisional regret or dissatisfaction in either the unadjusted or adjusted analysis (aRD, −6.04; 95% CI, −16.36 to 4.29 for completely successful vs unsuccessful/unsure; aRD, 0.02; 95% CI, −9.68 to 9.72 for intermediate vs unsuccessful/unsure).

## Discussion

In this secondary analysis of participants randomly assigned to receive antibiotics in the CODA trial, positive beliefs at baseline regarding the likelihood of success of antibiotics were associated with a lower risk of appendectomy within 30 days, with a marked effect within the first 48 hours. Participants’ beliefs were also associated with self-reported resolution of signs and symptoms by 30 days. Moreover, the magnitudes of some of these associations were large; those who believed that antibiotics could be completely successful had about half as many appendectomies after antibiotics in the first 30 days as those who believed that antibiotics could be unsuccessful or were unsure. We did not find evidence of an association of baseline beliefs with high regret or dissatisfaction with treatment in this analysis.

The mechanisms that underlie this observed association between patients’ beliefs and outcomes for appendicitis remain to be determined. Evidence from other conditions suggests biologic mechanisms contribute to the placebo effect, though mechanisms are thought to vary between conditions.^14^ For example, placebo effects in analgesia have been tied to endogenous opioid, dopamine, and endocannabinoid pathways related to expectations, conditioning, and reward learning.^15^ Analogous mechanisms have not been well described for infectious and inflammatory conditions. We propose several potential explanations for this effect in antibiotics for appendicitis that may direct further investigation. It is possible that participants’ beliefs about antibiotics may have led to differential antibiotics adherence behavior. Prior work has found that patients’ beliefs about and expectations for treatments can affect outcomes through medication adherence in conditions ranging from smoking cessation to HIV infection.^16,17^ Furthermore, appendectomy after antibiotics may not be determined strictly by physiologic factors. The assessment of the response to antibiotics may have had a subjective component, perhaps driven by participant-reported pain and other symptoms or clinician beliefs about what treatment is best. Indeed, the experience and reporting of pain have been previously shown to be associated with patients’ beliefs and expectations.^18^ It is possible that participants’ beliefs influenced the likelihood and/or extent to which they reported symptoms to clinicians, and we did find an association between baseline beliefs and self-reported resolution of signs and symptoms at 30 days. We did not survey clinicians about their reasons for performing an appendectomy, but participants’ reports of worsening pain may very well have motivated the decision for surgery.

### Limitations

There are important limitations to acknowledge. This was a secondary analysis of a clinical trial in which participants consented to randomization. Additionally, they were asked the beliefs question after viewing standardized informed consent materials articulating the known risks and benefits, as well as the uncertainty surrounding antibiotics treatment. Thus, those with beliefs that antibiotics were likely to be completely successful or unsuccessful represent intriguing groups that potentially either did not understand or think relevant the data about uncertainty presented in the video. Of note, in this sample there were very few individuals with a belief score less than 5; we therefore cannot comment on how a strongly negative belief might be associated with participants’ outcomes. Furthermore, it is not clear to what extent the associations observed in this randomized cohort would hold in groups not willing to randomize, not provided such formalized information about risk, or the population at large.

Additionally, the beliefs survey question was a nonstandard scale. Participants were classified into belief groups based on the response distribution and the intent to compare groups of reasonable size with one another. Furthermore, we did not investigate nor attempt to modify how participants interpreted the idea of treatment success, which may have been a source of heterogeneity in responses. It is also not clear to what extent the beliefs being measured may have been influenced by health care professionals or others with whom participants interacted in their initial care for appendicitis. In the adjusted analysis, we were limited to factors measured during the CODA trial, and the sample was too small to adjust for every potentially important factor; thus, there may be residual confounding contributing to the apparent association between beliefs and outcomes. Additionally, this study was neither intended nor powered to assess the question of what factors—individual or systemic—may affect patient beliefs, but our findings suggest that this may be an important area for further study. We excluded 351 participants from the CODA antibiotics group in this analysis because either (1) when answering the baseline survey, they were already aware of their treatment assignment, or (2) the timing of their survey responses relative to treatment assignment was not recorded. We felt this was necessary to measure preexisting beliefs, but this reduced the size of the cohort and limited the analysis. Although no clear differences were seen between the group analyzed in this study and all those assigned to the antibiotics group in the CODA trial, there may be unmeasured differences that limit the generalizability of these results.

## Conclusions

Findings of this secondary analysis of the CODA randomized clinical trial suggest that understanding patient beliefs about treatment success when making decisions about treatment for appendicitis are important. This information might be expected to improve shared decision-making, even as we acknowledge that beliefs may influence outcomes in ways not yet fully understood. The pathways relating beliefs to outcomes, the potential modifiability of beliefs to improve outcomes,^19^ and the interplay of patient, caregiver, and clinician beliefs are important areas for further investigation.

## Notes

Supplement 1. Trial Protocol

Supplement 2. Statistical Analysis Plan

Supplement 3. **eFigure.** Distribution of Beliefs in CODA Antibiotics Group **Appendix 1.** Description of Variables **Appendix 2.** Description of Multiple Imputation **Appendix 3.** Description of Propensity Scores **Appendix 4.** Complete Case Data **Appendix 5.** The CODA Trial Sites and Site Leads

Supplement 4. Nonauthor Collaborators

Supplement 5. Data Sharing Statement
