# Supplementary material for: Association of Patient Belief About Success of Antibiotics for Appendicitis and Outcomes: A Secondary Analysis of the CODA Randomized Clinical Trial
Source: JAMA Surg. 2022 Oct 5;157(12):1080–7. doi: 10.1001/jamasurg.2022.4765 (PMC9535504; doi:10.1001/jamasurg.2022.4765)
Supplement: Supplement 1. — Trial Protocol [file jamasurg-e224765-s001.pdf]

# The Comparison of Outcomes of Antibiotic Drugs and Appendectomy (CODA) Trial

Developed by the **C**ollaborative of **A**cute Care **S**urgery and **M**edicine (CASM) Trialist Group

Co-Directors David Talan, MD and David R. Flum, MD MPH

**Study Funder:**

Patient Centered Outcomes Research Institute (Principal Investigator-Flum)

**Version Number and Date:**

~~V1.0 – November 25, 2015~~

~~V2.0 – April 3, 2016~~

~~V3.0 – January X, 2017~~

~~V3.5 – April 3, 2016~~

~~V5.0 April 2017~~

~~V6.0 December 2018~~

V6.1 February 2019

**Table of Contents**

1.0 Key Personnel 6

2.0 Background and Rationale ....8

2.1 Rationale ....8

3.0 Study Aims and Methods ....10

3.1 Study Aims ....10

3.2 Methods ....10

4.0 Participant Screening and Enrollment ....11

4.1 Participant Screening ....11

4.1.1 Retrospective EMR review ....11

4.2 Inclusion Criteria ....12

4.3 Exclusion Criteria ....12

4.4 Consent Process ....13

4.4.1 Randomization Procedures ....14

4.4.2 Those who Decline Randomization ....14

4.5 Antibiotics Therapy Arm ....15

4.5.1 Discharge Criteria ....15

4.5.2 Guidance for Appendectomy in Patients Randomized to Antibiotics ....16

4.6 Appendectomy Arm ....16

4.7 Discharge Instructions ....16

5.0 Study Schedule Overview, Research Assessments, Medical Record Review, Call Triggers, Withdrawal, Compensation, and Retention ....17

5.1 Study Schedule Overview ....17

5.2 Research Assessments ....18

5.2.1 Baseline Assessment (Index Encounter) ....18

5.2.2 Study Enrollment Post-Discharge Call ....18
5.2.3 Week 1 and Week 2 Assessments ....19
5.2.4 Week 4 Assessment ....20
5.2.5 Follow-up Assessments (3, 6, 9, 12, 18, and 24 months) ....20
5.2.6 Optional Extended Follow-up Assessments (30, 36, 42, 48, 54, and 60 months) ....21
5.3 Medical Record Review ....21
Time points ....21
Data Collection ....22
5.4 Research Coordinator Call Triggers and Clinical Contact ....23
5.4.1 Early Call Triggers (Up to 3 Months) ....23
5.4.2 Late Call Triggers (Beyond 3 Months) ....23
5.4.3 Clinician Education ....24
5.5 Reasons for Withdrawal ....24
5.5.1 Handling of Withdrawals ....24
5.6 Compensation ....24
5.6.1 Participant Compensation ....24
5.6.2 Site and Site Lead Compensation ....24
5.7 Retention Activities ....25
5.7.1 Maintaining Contact ....25
5.7.2 Contact between Assessments ....25
6.0 Outcome Assessments ....25
6.1 Primary Outcome ....25
6.2 Secondary Outcomes ....26
6.2.1 Clinical Complications ....26

6.2.2 Eventual Appendectomy ........ 27

7.0 Data Management and Information Security ........ 27

7.1 Data Management ........ 27

7.1.2 Data Quality Monitoring ........ 27

7.2 Information Security ........ 28

8.0 Statistical Analysis Plan and Stopping Rules ........ 29

8.1 Randomization Overview ........ 29

8.2 Outcomes Measures ........ 29

8.3 General Analytic Strategy ........ 30

8.4 Missing Data ........ 32

8.5 Sample Size and Statistical Power ........ 32

8.6 Data and Safety Monitoring Plan (DSMP) ........ 33

9.0 Study Related SAEs ........ 35

9.1 Identification of SAEs ........ 35

9.2 SAE Reporting ........ 36

9.3 Assignment of SAEs as Treatment Related ........ 36

10.0 Reporting Procedures ........ 36

11.0 Quality and Performance Management Plans ........ 37

11.1 Quality Management Plan ........ 37

11.2 Performance Management Plan ........ 37

11.3 Protocol Violations and Deviations ........ 38

12.0 Protection of Human Participants ........ 39

13.0 Ancillary Studies ........ 40

14.0 References ........ 40

**1.0 Key Personnel**

| | |
|---|---|
| Principal Investigators | David R. Flum, MD MPH<br>University of Washington<br>Department of Surgery<br>1959 NE Pacific Street, Box 356410<br>Seattle, WA 98195<br>Phone: 206-616-5440<br>Email: daveflum@uw.edu |
| | David Talan, MD<br>Olive View-UCLA Medical Center<br>Department of Emergency Medicine<br>14445 Olive View Drive, North Annex<br>Sylmar, CA 91342<br>Phone: 818-364-3107<br>Email: dtalan@ucla.edu |
| Director, Data Coordinating Center | Patrick Heagerty, PhD<br>University of Washington<br>Department of Biostatistics<br>4333 Brooklyn Avenue NE, Box 359461<br>Seattle, WA 98105<br>Phone: 206-616-2720<br>Email: heagerty@uw.edu |
| Director, Clinical Coordinating Center | Giana H. Davidson, MD MPH<br>University of Washington<br>Department of Surgery<br>1959 NE Pacific Street, Box 356410<br>Seattle, WA 98195<br>Phone: 206-543-9559<br>Email: ghd@uw.edu |
| Director, Stakeholder Coordinating Center | Danielle Lavallee, PhD PharmD<br>University of Washington<br>Department of Surgery<br>1107 NE 45th Street, Box 354808<br>Seattle, WA 98105<br>Phone: 206-221-1799<br>Email: lavallee@uw.edu |

| | |
|---|---|
| Project Manager - Washington | Erin Carney<br>University of Washington<br>Department of Surgery<br>1107 NE 45th Street, Box 354808<br>Seattle, WA 98105<br>Phone: 206-685-9770<br>Email: ecarney@uw.edu |
| Project Manager - California | Anusha Krishnadasan, PhD<br>Olive View-UCLA Medical Center<br>Department of Emergency Medicine<br>14445 Olive View Drive, North Annex<br>Sylmar, CA 91342<br>Phone: 818-364-3111<br>Email: idnet@ucla.edu |

## 2.0 Background and Rationale

For over 100 years, surgical removal of the appendix to treat acute appendicitis has been one of the most commonly performed procedures, resulting in nearly $2 billion in healthcare costs annually.[1] Patients undergoing appendectomy miss a median of 10-14 days from work and only resume "normal" levels of activity 7-21 days after surgery.[2] As an alternative to surgery, the successful use of antibiotics-alone as a treatment for acute uncomplicated appendicitis (AUA) was first reported in 1959 in a large British case series.[3] As surgical procedures and anesthesia became safer, the antibiotics-only approach to appendicitis was usually restricted to patients with more complicated disease, usually followed by an interval appendectomy. The exception was patients who did not have access to prompt surgical services such as Naval submariners whose initial results were very satisfactory.[4,5]

Inspired by the antibiotics-only approach results between 1956 and 2015, and with advances in radiologic imaging to diagnose and stage the progression of disease, there were six randomized trials comparing antibiotics to appendectomy among patients with AUA. These trials, all performed in Europe,[6-11] found that nearly 3 out of 4 patients in the antibiotics treatment group avoided surgery in the first year after diagnosis. A meta-analysis of five of these studies (the most recent published in 2015) found that antibiotic treatment was associated with a 46% reduction in complications and less pain and disability than routine appendectomy.[12] No participant deaths occurred in the trials, and the rates of perforation were lower among those in the antibiotic treatment group. However, existing trials suffer from important limitations. These include small sample sizes leading to indeterminate results, lack of imaging leading to potential inclusion of patients with complicated appendicitis and potentially without appendicitis at all, inexact and treatment dependent outcome definitions (rate of appendectomy) and operation/re-operation criteria, arbitrarily prolonged hospitalization, limited use of laparoscopic techniques, inadequate antibiotic regimen activity, and short-term follow-up (no studies followed patients beyond one year). Furthermore, none of these trials included keeping a record of the patient's experience with treatment in a standardized fashion. Perhaps as a result of these evidence gaps, nearly all United States (U.S.) patients with appendicitis (97.5% according to a recent California [CA]-based administrative database analysis[13] and similar findings in Washington [WA] [personal communication DRF]) undergo appendectomy. Conversely, in Europe where the trials originated, it now appears that 1 in 5 patients with AUA are being offered the antibiotics approach.

It remains to be determined whether patient experience with the antibiotics strategy (performing appendectomy only when the patient gets worse on antibiotics or has a recurrence of disease) is "just as good as" appendectomy. This comparison should consider the overall impact of the treatment on their health, well-being, quality of life (QoL), healthcare burden, and all clinical endpoints (e.g., resolution of symptoms, need for eventual appendectomy, complications). It also remains to be determined what patient and clinical factors are associated with worse outcomes.

### 2.1 Rationale

The Comparison of Outcomes of Antibiotic Drugs and Appendectomy (CODA) trial aims to test if, from a patient's perspective, the antibiotics strategy is "just as good as" surgery. We believe that patients, clinicians, and the people who pay for healthcare (both patients and insurers) will find the antibiotics approach acceptable if 1) it results in high rates of treatment success, 2) does not increase complications or adverse events (AEs), and 3) provides an equivalent or better patient experience. A large-scale non-inferiority trial is expected to result in a change in

the management of one of the most common human illnesses. If non-inferiority is demonstrated (or superiority of the antibiotics approach identified), that finding will improve patient choice and should support a shift to the less invasive approach. If non-inferiority is not established, results may help to delineate the trade-offs between the two treatment approaches and inform decision-making.

The observation that patients with AUA can be cured with antibiotics-alone has a scientific rationale. Traditionally, appendicitis was thought to result from a blockage of the appendix and that, if left untreated, inevitably led to a perforation. Contrary to this physiologic model, Carr demonstrated that obstruction of the appendix is an unlikely primary cause in the majority of patients and that most are caused by an enteric infection.[14] A recent randomized trial found an increased rate of appendectomy with early use of computed tomography (CT) imaging and diagnostic laparoscopy, but case reports of appendicitis remission documented by serial CT all suggest that leaving the appendix in place does not inexorably lead to clinical compromise.[15] A concern of clinicians when considering treating AUA with antibiotics is that not removing the appendix may put patients at risk for a more complicated operation and higher morbidity. Evidence suggests, however, that perforated appendicitis is a pre-hospital and pre-antibiotic event and that non-perforated appendicitis is a “different” disease. This theory is supported by a general lack of relation between a delay in surgery of up to 24-36 hours among patients with appendicitis who are treated with antibiotics and perforation rates.[16,17] Most recently, *Fusobacterium sp.*, a genus of enteric Gram-negative anaerobic bacteria, rather than an obstructive stone, was found to be correlated with the presence of appendicitis and the degree of inflammation.[18] What remains to be determined is whether certain bacterial colonies or features of individual immune response are most associated with progression of appendicitis and whether successful outcomes for people undergoing antibiotic management can be predicted based on the patient’s characteristics.

An additional rationale for this study is to address limitations of prior trials. To avoid misclassification problems of other trials and consistent with U.S. norms,, all patients will undergo standard radiographic imaging including CT, ultrasound (US), or magnetic resonance imaging (MRI). Patients with an appendicolith will be included in the trial but considered a unique subgroup and will be evaluated as part of a pre-specified analysis (potentially excluding them from future recruitment if an early analysis demonstrates futility related to the primary antibiotic approach not being successful). To reflect usual and emerging techniques in treatment, the study includes both types of appendectomy (open and laparoscopic) and a broad range of antibiotic strategies including the option for an “all outpatient” treatment schedule with once daily dosing of longer-acting agents. Patients in the antibiotics arm will be given a minimum of 24 hours of intravenous (IV) antibiotics (using any appropriate dosing schedule and based on the patient’s ability to tolerate oral medication), followed by oral antibiotics for a total of 10 days of antibiotic treatment). Discharge from the hospital or emergency department (ED) or a change in treatment arm will be guided by clinical targets and reasons for change in treatment arms will be assessed. Participants will be followed for up to two years to assess for longer-term complications, eventual appendectomy (performed anywhere), QoL, gastrointestinal symptoms, and decisional regret. To quantify selection bias and to promote generalizability, all patients approached for the study and those who refuse randomization will be characterized at baseline. A parallel cohort of patients who refuse randomization (up to 250 who initiate the antibiotics strategy and 250 who select to undergo the appendectomy strategy) will be surveyed for up to two years. Participants in all cohorts will be given the option to participate in an extended follow-up that will assess long term complications, eventual appendectomy, QoL, and gastrointestinal symptoms.

## 3.0 Study Aims and Methods

### 3.1 Study Aims

Aim 1. Compare patient reported outcomes (PROs) in patients randomized to the antibiotics or appendectomy strategy.

Sub Aim 1. Compare PROs in patients without appendicolith randomized to the antibiotics or appendectomy strategy.

> Exploratory Aim A. Assess the rate of eventual appendectomy after starting the antibiotics treatment regimens in the first week, early (1-4 weeks) and late (2-24 months) periods and identify patient clinical characteristics (e.g., appendicolith) as well as clinician and practice site characteristics associated with eventual appendectomy in the antibiotic therapy group.

Aim 2. Compare clinical outcomes in patients randomized to antibiotics versus appendectomy.

Sub Aim 2. Compare clinical outcomes in patients without appendicolith randomized to the antibiotics or appendectomy strategy.

> Exploratory Aim B. Compare randomized patients to those in a concurrent observational cohort to identify selection characteristics and outcome differences between the two groups.

The central hypotheses of the CODA trial is that PROs among patients in the antibiotics arm will not be inferior to those in the appendectomy arm and that adverse clinical outcomes will not be significantly worse (non-inferior) among patients being treated with antibiotics.

### 3.2 Methods

This will be a multi-site, open trial in which participants with AUA are randomized to one of two initial treatment strategies, appendectomy or antibiotics (with appendectomy only for those with a deteriorating clinical condition while being treated with antibiotics or disease recurrence).

The University of Washington's (UW) Surgical Outcomes Research Center (SORCE) will lead a Clinical Coordinating Center (CCC) and Stakeholder Coordinating Center (SCC). The UW Center for Biomedical Statistics (CBS) will serve as the Data Coordinating Center (DCC).

The CCC will coordinate all aspects of the trial. Investigators at SORCE will coordinate the study at the University of Washington Medical Center (UWMC) and up to twenty other clinical sites. Investigators at Olive View-UCLA Medical Center in CA will coordinate the study there and up to four other clinical sites. In preparing for the study, 18 potential hospital sites were engaged and up to 20 will be considered for involvement in the study, based on readiness of infrastructure, staff engagement, patient population characteristics, and number of appendicitis cases.

Prior to study initiation and enrollment, all clinicians and staff who encounter patients with possible appendicitis will complete study-specific training including demonstrating successful use of the antibiotics strategy in five patients and site experience recorded in a non-protected health information (PHI) based registry (www.certain.org/appysurvey) or log reviewed with the study leadership team. All staff engaging patients with appendicitis at all study sites will undergo online and in-person educational and orientation sessions for the topic and the study protocol.

Table 1. Potential Study Sites, Characteristics, and Projected Yearly Volume of Appendicitis.

| Medical Center | Total n/yr | N (%) | | | | | | | |
|---|---|---|---|---|---|---|---|---|---|
| | | Female | > 65 Years | Medicaid | Hispanic | Black | Asian | AI/AN* | Pacific Islander |
| Harborview | 47 | 13 (27.7) | 2 (4.3) | 12 (25.5) | 12 (25.5) | 7 (14.9) | 7 (14.9) | 2 (4.3) | 4 (8.5) |
| Madigan Army | 107 | 33 (53.6) | 16 (15.0) | 0 (0.0) | 4 (3.7) | 4 (3.7) | 2 (1.9) | 0 (0.0) | 1 (0.9) |
| Northwest | 77 | 36 (46.8) | 14 (18.2) | 8 (10.4) | 2 (2.6) | 6 (7.8) | 6 (7.8) | 0 (0.0) | 0 (0.0) |
| Providence | 294 | 147 (50.0) | 54 (18.4) | 47 (16.0) | 26 (8.8) | 6 (2.0) | 16 (5.4) | 5 (1.7) | 3 (1.0) |
| Sacred Heart | 180 | 91 (50.6) | 35 (19.4) | 18 (10.0) | 2 (1.1) | 4 (2.2) | 2 (1.1) | 4 (2.2) | 1 (0.6) |
| Skagit Valley | 93 | 57 (61.3) | 21 (22.6) | 17 (18.3) | 20 (21.5) | 3 (3.2) | 1 (1.1) | 2 (2.2) | 0 (0.0) |
| Swedish | 386 | 179 (46.4) | 67 (17.4) | 20 (5.2) | 35 (9.1) | 11 (2.9) | 39 (10.1) | 0 (0.0) | 3 (0.8) |
| University of Washington | 85 | 38 (44.7) | 14 (16.5) | 12 (14.5) | 8 (9.4) | 1 (1.2) | 10 (11.8) | 2 (2.4) | 0 (0.0) |
| Virginia Mason | 87 | 41 (47.1) | 26 (29.9) | 2 (2.3) | 4 (4.6) | 3 (3.5) | 8 (9.2) | 1 (1.2) | 2 (2.3) |
| Olive View-UCLA | 221 | 85 (38.5) | 2 (0.9) | 173 (78.3) | 183 (82.8) | 2 (0.9) | 6 (2.7) | 0 (0.0) | 4 (1.8) |
| Harbor-UCLA | 263 | 112 (42.6) | 4 (1.5) | 116 (44.1) | 229 (87.1) | 19 (7.2) | 8 (3.0) | 0 (0.0) | 0 (0.0) |
| Los Angeles County+USC | 360 | 148 (41.1) | 23 (6.4) | 133 (36.9) | 236 (65.6) | 54 (15.0) | 21 (5.8) | 1 (0.3) | 1 (0.3) |
| All Sites | 2200 | 980 (44.5) | 278 (12.6) | 558 (25.4) | 761 (34.6) | 120 (5.5) | 126 (5.7) | 17 (0.8) | 19 (0.9) |

## 4.0 Participant Screening and Enrollment

### 4.1 Participant Screening

Patients presenting to the ED will be screened by study investigators and/or study coordinators seven days a week based on alerts from clinicians, staff, or screening of ED logs. Patients will be identified as potential candidates for the study based on inclusion and exclusion criteria collected as part of standard care, including confirmatory diagnostic imaging (CT, US, and/or MRI).

The CODA Screening Form must be completed for all patients ≥18 years of age presenting to the ED (hospital/clinic) with presumptive diagnosis of appendicitis that has not been previously treated. Completion and data-entry of this form is required regardless of patient eligibility to ensure a complete description of the population of interest. Those who do not meet eligibility criteria or have exclusion criteria present will be considered ineligible. Additionally, this form must be completed and entered for patients that were not approached.

Sites are responsible for maintaining their own screening log in accordance with their institutional policies and guidelines. This log should document adequate information such that the research team may disclose to the institutional compliance department that a patient's electronic medical record (EMR) was accessed to screen for potential eligibility for the CODA trial. UW Medicine requires disclosure of accessing the EMR for purposes other than treatment, payment, and health care operations.

Sites are required to provide a monthly de-identified appendicitis census to the UW CCC. This census will be compared to the number of participants reported as screened at each site and will serve as a measure of protocol compliance.

#### 4.1.1 Retrospective EMR review

For patients excluded due to concern for appendiceal cancer or mass, an EMR review that is separate from the scheduled CODA participants EMR review will be completed. This EMR review may be done retrospectively and must be done in accordance with site Institutional Review Board (IRB) recommendations and approvals. The intention of this EMR review is to

fully characterize this particular patient population and to better understand whether or not the CT reading appropriately excludes patients.

EMR review for these patients will include review and abstraction of the following patient records: final pathology report, operative report, radiology images and reports that indicate association with cancer.

EMR review will be completed for patients excluded for the following reasons: 1) appendiceal soft-tissue mass, 2) imaging features of mucocele or tumor, 3) concern for any carcinomatosis on imaging, or 4) qualitative reason indicative of concern for cancer or mass.

Sites will be provided with a list of screening IDs for patients who were ineligible due to the aforementioned reasons.

### 4.2 Inclusion Criteria

1. Adult ≥18 years;
2. First clinical diagnosis of AUA established by clinical care team, supported by any of the following usual care radiological tests (CT, US, and/or MRI). AUA is defined by the usual signs, symptoms, and imaging finding of appendicitis **without**:
    a. Diffuse peritonitis on clinical exam (i.e., rigid abdomen / four quadrant peritonitis);
    b. Radiologic findings of :
        i. Free air;
        ii. Walled off fluid collection concerning for an abscess;
        iii. Significant amounts of intra-abdominal fluid throughout abdomen (i.e., more than trace fluid); or
        iv. Extent of inflammation or adjacent organ involvement on radiologic imaging such that urgent appendectomy is relatively contraindicated or not intended.
3. Ability to provide written informed consent in English or Spanish.

### 4.3 Exclusion Criteria

Participants must not have any of the following exclusion criteria:

1. Unable or unwilling to return or be contacted for and/or research surveys;
2. Currently incarcerated in a detention facility or in police custody (patients wearing a monitoring device can be enrolled) at baseline/screening;
3. Evidence of severe sepsis or septic shock (e.g., new presumed sepsis-related organ dysfunction, elevated lactate, and/or fluid unresponsive hypotension); these patients can be enrolled if signs of sepsis resolve with fluid resuscitation and pain medication administration while in the ED;
4. Immunodeficiency (e.g., absolute neutrophil count <500/mm$^3$, chronic immunosuppressive drugs (e.g., oral corticosteroids, anti-TNF agents), or known AIDS [i.e., recent CD4 count <200 ] assessed by patient history);
5. Uncompensated liver failure;

6. Taking medication to treat active inflammatory bowel disease (e.g., Crohn’s, ulcerative colitis);
7. Active treatment for malignancy, not in remission (patients undergoing active chemotherapy or with plans for chemotherapy in the following 30 days are excluded);
8. Pregnant or expectation of becoming pregnant in the 30 days following baseline/screening;
9. Expected concurrent hemodialysis, peritoneal dialysis, or treatments using indwelling venous catheters or at risk for bacterial seeding of implants;
10. Recent (within 90 days) placement of surgical implant (e.g., pacemaker, joint prosthesis, mechanical valve);
11. Indwelling Left Ventricular Assist Device (LVAD);
12. Patients with another infection (e.g., pneumonia, urinary tract infection) that requires treatment with another antibiotic at baseline/screening;
13. Concurrent illness that would otherwise mandate hospitalization outside of appendicitis and associated symptoms at baseline/screening;
14. Imaging findings of any of the following that would make the patient ineligible for one arm of the trial:
    a. Appendiceal soft-tissue mass;
    b. Imaging features of mucocele or tumor concerning for malignancy of the appendix or in other organs; or
    c. Concern for any carcinomatosis on imaging;
15. Severe allergy or reaction (e.g., immediate urticaria or anaphylaxis) to <u>all</u> of the proposed antibiotics (See Section 4.5);
16. Prior enrollment in the study or other investigational drug or vaccine while on study treatment;
17. Abdominal/pelvic surgery in the past month; or
18. More than seven hours have transpired since the patient received the first parenteral dose of antibiotics.

### 4.4 Consent Process

The research coordinator and a representative from the medical team will confirm the patient’s eligibility for the study based on inclusion and exclusion criteria. A research team member will approach all eligible patients and ask them to review an informed decision making video using a handheld device. The English or Spanish-language video explains both treatment options and the research study using linguistically and culturally appropriate language. In the event that the tablet is malfunctioning or the patient declines to watch the video, the research coordinator will provide an informational pamphlet that includes the same content as the video. There are two versions of the video and pamphlet, one focused on positive messaging, the other negative. Participants who present on odd calendar days will be shown Version 1; those who present on even days will be shown Version 2. Which version was shown to the patient will be documented on the screening form in order to determine whether phrasing outcomes in different ways impacts subject participation. The video and informational pamphlet both conclude with a

request of the patient to read and sign an informed consent document for participation in the randomization cohort.

a. For patients consenting to randomization, they will be randomly assigned to either the antibiotics or appendectomy arm immediately following consent and assigned a study ID;
b. Patients refusing consent to randomization may first be invited to participate in the observational cohort, and if that is declined, may then be offered participation in the EMR review cohort. This will be done by separate consent(s) with the participant selecting one of two participation options:
    - Option 1. Observational cohort (no randomization). Participants selecting the observational cohort will be asked to complete the same research assessment activities as the randomized cohort and will have their appendicitis treated following the same treatment protocols.
    - Option 2. EMR review cohort. Participants selecting this option will be followed by passive EMR review only. They will not be contacted again for study purposes and will not be asked to complete the Baseline Assessment or the Follow-Up Assessments described in Section 5.2.

In summary, depending on the site, there will be two or three consents for this study with the option to participate in the following groups: 1) randomization cohort, 2) observational cohort, and 3) EMR review cohort. Participants may only consent to one study group. The randomization consent will be presented to the patient following the informed decision-making video or material presentation. Patients declining randomization will then be presented with a different consent form to consider participation in the observational and EMR review only cohorts.

Consenting participants will also need to complete a Health Insurance Portability and Accountability Act (HIPAA) form in accordance with their institutional guidelines. For some partnering sites, HIPAA language will be incorporated into the informed consent document but for others, this will be a separate document. For example, the UW requires that a participant sign a separate HIPAA document to indicate consent that their EMR is accessed and reviewed for research purposes. The HIPAA form is not reviewed by the UW's Human Subjects Division but is required per UW Medicine policy.

#### 4.4.1 Randomization Procedures

After informed consent, participants will be randomized to appendectomy or to antibiotics using computer generated assignments. Randomization will be based on a random number sequence, stratified by site and presence of appendicolith, and will also be blocked to assure balance across treatment groups. Randomization failures (defined as a patient who agreed to randomization but whose treatment was allocated in a non-random fashion or was randomized under inaccurate information) will be recorded as protocol violations and these consenting participants will continue to be followed. Their outcomes will be recorded and examined in a separate sub-analysis.

#### 4.4.2 Those who Decline Randomization

Depending on the site, participants who decline randomization will be approached to provide consent to the observational cohort. As many as 500 will be consented into an observational

cohort study. Recruitment will be capped by treatment choice, site location, and time. Once the cap has been reached, participants will be offered participation in the EMR review only cohort.

### 4.5 Antibiotics Therapy Arm

Patients in the antibiotics arm will receive a total of 10 days of antibiotics, with a minimum of 24 hours using an IV antibiotic formulation (administered in q8, q12, or q24 hour regimens with or without concurrent oral antibiotics) followed by oral antibiotics for the remainder of the 10 days. Patients will be offered a treatment regimen of antibiotics based on guidelines published jointly by the Surgical Infection Society and the Infectious Disease Society of America.[19] The first dose of antibiotics will be given in the ED at the time of diagnosis of appendicitis for both treatment arms. This flexibility in antibiotics choice is not expected to impact outcome but should improve the generalizability of the results, and therefore, implementation of the findings. Any of the IV antibiotics options (*Single antibiotic*-Cefoxitin, Ertapenem, Moxifloxicin, Tigecycline, Ticarcillin-Clavulanic Acid or *Dual antibiotics*-Metronidazole or Clindamycin plus one of the following-Cefazolin, Cefuroxime, Ceftriaxone, Cefotaxime, Ciprofloxacin, Levofloxacin) will be considered acceptable. At many sites, these will be administered to inpatients only. For clinicians and patients interested in outpatient receipt of antibiotics, the available options will include Ertapenem or Moxifloxacin (q24) alone or Ceftriaxone (IV) plus IV or oral Metronidazole or Clindamycin, as tolerated. Although these antibiotics are considered acceptable and equally efficacious for intra-abdominal infections by the aforementioned professional societies, outcomes based on antibiotics used and antibiotics regimen will be monitored at regular intervals by the DCC and reported to the Data and Safety Monitoring Board (DSMB) for consideration in protocol modification. After IV antibiotics are administered for a period of at least 24 hours, a regimen of oral antibiotics will be continued to complete a total treatment length of 10 days. Acceptable oral regimens should be based on *in vitro* activity against aerobic and anaerobic Gram-negative bacteria known to cause appendicitis, and practical experience with oral antibiotic regimens used to treat diverticulitis. Preferred oral antibiotic regimens include Moxifloxacin alone, and combinations such as Metronidazole or Clindamycin, for anaerobic bacteria coverage, plus Ciprofloxacin or Levofloxacin, or an oral Cephalosporin for aerobic Gram-negative bacteria coverage. Antibiotics will be prescribed by clinicians and procured by patient as per usual clinical care.

#### 4.5.1 Discharge Criteria

Beginning after adequate evaluation of a patient following treatment, clinical staff will have the option to discharge the patient if they meet specific criteria:

1. Stable and near normal vital signs appropriate for age;
2. Afebrile;
3. Signs and symptoms controlled with oral analgesics;
4. Patient able to tolerate oral fluids (e.g., water, broth) and medication;
5. Patient and clinician (emergency physician and surgery team agreement) report that discharge is acceptable; and
6. Routine clinical follow-up visit confirmed.

#### 4.5.2 Guidance for Appendectomy in Patients Randomized to Antibiotics

For participants randomized to the antibiotics arm, appendectomy will be recommended when there is:

1. Development of diffuse peritonitis;
2. Development of severe sepsis/septic shock (e.g., new presumed sepsis-related organ dysfunction, elevated lactate, and/or fluid unresponsive hypotension)and/or
3. Worsening signs and symptoms of appendicitis after 48 hours following the first antibiotics dose.

Absent these criteria, patients will be encouraged to continue with the antibiotics treatment strategy, especially during the first 48 hours of treatment. At any point after initiating the antibiotics strategy, the surgeon and patient can determine that an appendectomy should be performed. When a decision for appendectomy is being considered, the site lead investigator will be notified to determine if any of the above criteria are met or, if not, to review the surgeon's and patient's specific reasons for recommending (surgeon) or requesting (patient) appendectomy. These reasons will be recorded for analysis and reviewed by the project manager. If the project manager believes an appendectomy was recommended by the surgeon outside of the protocol, it will be reviewed by a clinician not otherwise affiliated with the study who will follow up with the site PI. All appendectomies performed outside of study protocol, other than those requested by the participant, will be reported as protocol violations.

All participants will be encouraged to return to the hospital or clinic site if they have recurrent signs and symptoms of appendicitis after the four-week assessment and encouraged to undergo repeat diagnostic imaging to detect recurrent appendicitis. Participants in the antibiotics arm who return to any of the study sites during the follow-up period (but after the four-week assessment) with recurrent appendicitis will be offered appendectomy or the option of another attempt at antibiotics but will not be re-randomized or moved to another cohort.

### 4.6 Appendectomy Arm

All patients randomized to appendectomy will receive one dose of antibiotics per currently accepted standards described in Section 4.5 when diagnosis is confirmed in the ED. Patients may also receive preoperative antibiotics per hospital standards for surgical infection prevention bundle.

Appendectomy will be performed by an open or laparoscopic approach, depending on patient and surgeon preference.

### 4.7 Discharge Instructions

The same criteria for discharge will be applied to the antibiotics group and the appendectomy group (Section 4.5.1) including standard discharge instructions and return precautions, telephone or in-person appointments for usual clinical care follow-up (one to three weeks after discharge), and contact information for the study coordinator and surgeon or emergency medicine investigator at that site. Participants will also be provided with 24-hour hospital/clinic paging information for a clinician at their site should they have any emergent clinical concerns.

# 5.0 Study Schedule Overview, Research Assessments, Medical Record Review, Call Triggers, Withdrawal, Compensation, and Retention

## 5.1 Study Schedule Overview

Consenting participants will be asked to complete research assessments on the topics and at the time points described in Table 2. Site research coordinators (RCs) will oversee the completion of the Baseline Assessment in-person and will also contact participants by phone to complete the Week 1 and Week 2 Assessments. Participants will then be contacted by phone, mail, or email by the UW study staff to complete the Week 4 Assessment, Quarterly Assessments for the first year, and Biannual Assessments in the second year after the participant's initial ED presentation.

Participants will be given the option to consent to extended follow-up beyond the two-year mark. These participants will be surveyed annually for another 3 years for a total follow-up period of 5 years.

Table 2. Participant Assessment Schedule.

| **Item** | **Baseline** | **Follow-Up Time Point** | | | | | | | | | **Optional Extended Follow-up** | | |
|---|---|---|---|---|---|---|---|---|---|---|---|---|---|
| | | **First 4 Weeks** | | | **Month** | | | | | | **Month** | | |
| | | **1** | **2** | **4** | **3** | **6** | **9** | **12** | **18** | **24** | **36** | **48** | **60** |
| Patient Point of Contact | **Site RC** | **Site RC** | | **UW study staff (Site RC as backup)** | | | | | | | **UW study staff** | | |
| Contact Information | x | x | x | x | x | X | x | x | x | x | x | x | x |
| EQ-5D[20] | x | | | x | x | X | x | x | x | x | x | x | x |
| 10-PROMIS Global Health Short Form[21] | x | | | x | x | | | x | x | x | x | x | x |
| PROMIS-Pain Intensity | x | x | x | | | | | | | | | | |
| Symptom Onset | x | | | | | | | | | | | | |
| Beyond Demographics* | x | | | | | | | | | | | | |
| Treatment Satisfaction/Expectation | x | | | x | x | | | | | | | | |
| Gastrointestinal Quality of Life (GIQLI)[22] | | | | x | x | | | x | x | x | x | x | x |
| Healthcare Utilization | | x | x | x | x | X | x | x | x | x | x | x | x |
| Signs & Symptoms of Appendicitis | | x | x | x | x | X | x | x | x | x | x | x | x |
| Adverse Events | | x | x | x | x | X | x | x | x | x | x | x | x |
| Decision Regret Scale[23] | | | | x | x | | | x | | | | | |
| Major Life Changes | | | | x | x | X | x | x | x | x | x | x | x |
| Work Productivity Index | | x | x | x | x | | | | | | | | |
| Return to Work Information | | x | x | x | x | | | | | | | | |
| Medication Use | | x | x | x | x | | | | | | | | |
| Treatment Strategy Change | | x | x | x | | | | | | | | | |

*Includes the following topics: Demographics & Gender Identify, Caregiver Role, Instrumental Support, Employment/Student Status, Income, Pain Catastrophizing, Health Literacy, Social Support, Confidence in Treatment Success, Trust in Healthcare

To ensure successful retention of participants and completion of research assessments through the follow-up period, detailed contact information including name, address, phone numbers, email addresses, as well as alternate contact persons and phone numbers will be requested from participants at baseline (and updated at subsequent time points). While complete contact information is ideal to optimize future retention, only one means of contacting the participant is required for enrollment. For participants with upcoming travel plans abroad or who may live internationally, it is preferred that they provide phone and email contact information as mailed contact is slow and costly for the short windows around each research assessment.

The Week 1 and Week 2 follow-up assessments will be conducted by the site RCs. All subsequent follow-up assessments (Week 4 through the end of the follow-up period) will be managed and conducted by the UW study staff. The UW study staff are located in Seattle, WA. The UW study staff specialize in operationalizing multi-modal participant outreach and has dedicated research staff to support research studies conducted by UW investigators. For each timepoint, if a completed survey has not been received at the end of the standard outreach, UW staff and/or the site research coordinators will make additional outreach attempts.

### 5.2 Research Assessments

#### 5.2.1 Baseline Assessment (Index Encounter)

For all participants consenting to the randomized or observational cohorts, the research coordinator will support the participant as they complete the Baseline Assessment described in Table 2 (above). The Baseline Assessment should be completed in-person and ***prior to revealing the participant's random treatment assignment;*** *however, research coordinators are asked to document when the participant became aware of their assignment, should it be revealed prior to completion of the Baseline Assessment*. Survey questions will be presented in the order of importance in the event that the participant is unable to complete the full battery of questionnaires. The research coordinator will also extract case report data from the medical record related to the index hospital visit.

#### 5.2.2 Study Enrollment Post-Discharge Call

Twenty-four to forty-eight hours after enrollment a member of the research team will attempt to contact all participants to determine their current health status, answer any questions about the study, and to review the survey protocol. All patients will be encouraged to attend a usual care, in-person, or phone-based follow-up clinical assessment within two weeks of discharge. This clinic or phone-based contact with clinical staff is considered part of usual care and is not a research assessment.

At the time of enrollment and at the 48-hour contact, all participants will be given the study coordinator's contact information and will be encouraged to contact them with any questions or concerns. Participants will also be reminded of the 24-hour hospital/clinic paging information for a clinician at their site should they have any emergent clinical concerns.

Participants will be instructed at enrollment to contact the clinical team for return to clinic or the ED if they experience signs and symptoms of a complication. They will be evaluated by the site

lead clinician or their designee if they present to the ED or clinic for an unscheduled visit related to appendicitis at any time during the study period.

No in-person visits beyond those related to usual clinical care will be required as part of this protocol. As such, research-related clinical care charges will not be generated.

### 5.2.3 Week 1 and Week 2 Assessments

Participants will be asked about the following at Weeks 1 and 2:

1. Treatment strategy change;
2. General clinical follow-up;
3. Self-reported complications and AEs, including diarrhea;
4. Medication use, specifically:
   a. Pain medications (to estimate morphine equivalent dose used); and
   b. Number of antibiotic doses that were taken and number of remaining pills;
5. Return to work/school/regular activities;
6. Healthcare utilization including:
   a. Details on hospitalizations, clinic, standard of care healthcare visits, and ED visits and estimated time spent in healthcare;
   b. Use of repeat imaging during follow-up;
   c. Signs and symptoms of recurrent appendicitis;
   d. Appendectomy during follow-up period and reason (recurrence or elective/interval); and
   e. Out of pocket expenses.

Research coordinators will contact participants by phone to complete the Week 1 (contact window = days 7-10) and Week 2 (contact window = days 14-17) Assessments. (Day 0 is the day of study enrollment). Research coordinators will initiate outreach by phone on the day the window opens for each assessment period. If necessary, up to two calls may be made each day. These calls will be made at varying times of the day in an effort to reach the participant (e.g., morning or mid-day and again, after business hours).

If the contact protocol is exhausted and participant contact is not made, the corresponding assessment will be missed and attempts to contact the participant will resume as per contact protocol at the next assessment time point.

During the Week 2 Assessment, research coordinators will remind participants that they will be contacted by UW study staff to complete the remainder of their research assessments. Participants will be reminded that they are welcome to contact their research coordinator at any time if any questions or concerns should arise. UW study staff will communicate with the research coordinator to ensure appropriate reporting is completed should an AE or complication be reported to them.

### 5.2.4 Week 4 Assessment

In addition to the measures noted in Section 5.2.1, the following additional measures will be collected at the Week 4 Assessment (day 26-day 56):

1. European Quality of Life-5 Dimensions (EQ-5D) Quality of Life;[20]
2. 10 Patient Reported Outcomes Measurement Information System (PROMIS) Global Health Short Form;[21]
3. Decision Regret Scale;[23]
4. Gastrointestinal Quality of Life Index (GIQLI);[22]
5. Work Productivity Index; and
6. Treatment satisfaction.

Self-reported complications or AEs will be recorded. Any serious adverse events (SAEs) will be communicated to the relevant site study team to ensure appropriate reporting is completed.

UW study staff will contact the participant to complete the Week 4 Assessment. UW study staff will initiate a contact protocol approximately 26 days after the participant's initial ED presentation. This protocol will include a combination of phone, mail, and email as determined by the contact information provided by the participant. If the contact protocol is exhausted and the assessment has not been completed, the participant's research coordinator will be notified. They will review the EMR and provide any site-specific or EMR-specific updates that may be helpful; for example, an updated telephone number. Additional contact attempts (calls, emails, texts, etc) will be scheduled . These contact attempts will be made at varying times of the day in an effort to reach the patient (e.g., morning, mid-day, after business hours) and may be made by either the UW staff or the site staff. If the assessment is still not completed, it will be considered missed and attempts to contact the participant will resume as per contact protocol at the next assessment time point.

### 5.2.5 Follow-up Assessments (3, 6, 9, 12, 18, and 24 months)

At regular quarterly intervals through 12 months, then at 18 months and 24 months, the following will be assessed using phone, mail, or web-based surveys:

1. Complications, signs and symptoms related to appendicitis and related healthcare utilization, time spent in healthcare, time away from work/school, out of pocket expenses (3, 6, 9, 12, 18, 24 months);
2. Work Productivity Index (3 months);
3. EQ-5D[20] and 10-PROMIS[21] (3, 6, 9, 12,18, 24 months);
4. GIQLI[22] (3,12,18, 24 months); and
5. Decision Regret Scale[23] (3,12 months).

UW study staff will contact the participant to complete the quarterly and biannual assessments. UW study staff will initiate a contact protocol approximately seven days before the time point date. For example, outreach for the 3 month assessment for a participant who presented to the ED on May 1$^{st}$ (05/01) will begin on July 25$^{th}$ – seven days before August 1$^{st}$ (08/01). The protocol will be conducted over the course of approximately 3 weeks. This protocol will include a combination of phone, mail, and email as determined by the contact information provided by the

participant. If the contact protocol is exhausted and the assessment has not been completed, the participant's research coordinator will be notified. They will review the EMR and provide any site-specific or EMR-specific updates that may be helpful; for example, an updated telephone number. Additional contact attempts (calls, emails, texts, etc) will be scheduled. These contact attemptswill be made at varying times of the day in an effort to reach the patient (e.g., morning, mid-day, after business hours) and may be made by either the UW staff or the site staff. If the assessment is still not completed, it will be considered missed and attempts to contact the participant will resume as per contact protocol at the next assessment time point.

#### 5.2.6 Optional Extended Follow-up Assessments (36, 48, and 60 months)

At regular intervals through 60 months, the following will be assessed using phone, mail, or web-based surveys:

1. Complications, signs and symptoms related to appendicitis and related healthcare utilization, time spent in healthcare, out of pocket expenses;
2. EQ-5D[20] and 10-PROMIS[21]; and
3. GIQLI[22].

UW study staff will contact the participant to complete the annual assessments. Outreach will be conducted over the course of approximately 3 weeks and will include a combination of phone, mail, and email as determined by the contact information provided by the participant. If the contact protocol is exhausted and the assessment is not completed, additional phone calls will be scheduled. If the assessment is still not completed, it will be considered missed and attempts to contact the participant will resume as per contact protocol at the next assessment time point.

#### 5.2.7 Participant Burnout and Optimizing Data Collection

Participants may experience research assessment burnout due to the frequency and number of questions asked of them. To optimize complete data collection, research personnel should recognize a participant's reluctance to complete the research assessment and request that the participant instead complete a sub-set of survey responses (minimal research assessment). In the event that a participant prefers to complete a minimal research assessment, research personnel should prioritize asking the research participant questions related to signs and symptoms of appendicitis and aim to complete the EQ-5D survey. Study staff will be provided with a prioritization model to clarify what questions are high versus medium priority. The RC should use their best judgment to determine how many additional survey questions should be asked. It is preferred to miss some responses at one research assessment time point rather than risk the participant withdrawing altogether due to research assessment burnout.

### 5.3 Medical Record Review

#### Time points

Patients consenting to the study will have their EMR reviewed at (1) pre-determined time points and (2) ad-hoc time points if triggered by a survey response. RCs will conduct an EMR review to assess the following:

1. Complications for patients using the National Surgical Quality Improvement Program (NSQIP) definitions and protocol as well as those complications related to antibiotic use; and
2. Healthcare utilization events since index hospital discharge. Details of all healthcare utilization events related to appendicitis will include the following information:
    a. Length of stay;
    b. Use and results of repeat imaging; and
    c. Surgery type, if applicable.

The 3 cohorts will undergo pre-determined review at the following time points:

| | Randomized & Observational Cohorts | EMR Only Cohort |
|---|---|---|
| Index Visit | X | X |
| Week 1 | X | |
| Week 2 | X | |
| Week 4 | X | X |
| Year 1 | X | |
| Year 2 | X | X |

As needed, ad-hoc chart review will be conducted if a patient reports an adverse event and further documentation is needed.

When possible, participants will continue to have their EMR reviewed at Year 3, Year 4, and Year 5.

**Data Collection**

Data is collected using the following forms:

| Form | Description |
|---|---|
| Form Information | Captures information about when the form was filled out (time point), and by whom |
| ED Visit Form | Captures information about the hospital visit (demographics, admit/discharge times, symptoms) |
| Imaging Form | If patient had imaging done at any time point, captures information about the radiology tests and their findings |
| Laboratory Results Form | If patient had lab work done at any time point, captures information about the lab tests and their results |
| Index Visit Treatment Form | Filled out at the index visit only, captures information about randomization and/or cohort designation |
| Appendectomy Form | If patient had an appendectomy at any time point, captures information about the surgical procedure and treatment decision-making |
| Pathology Form | If patient had an appendectomy at any time point, captures information about the pathology results |
| Antibiotics Form | If patient was treated with antibiotics, captures information about the antibiotic treatment (both inpatient and at discharge) |
| Adverse Events Form | Captures information about adverse events at any time point |
| Discharge Form | Captures information about discharge status at any time point |

| | |
|---|---|
| Follow-Up Form | For scheduled EMR reviews, captures information about the review period including follow-up appointments, additional visits to the ER, etc. |

### 5.4 Research Coordinator Call Triggers and Clinical Contact

If the patient develops concerning signs or symptoms, the designated site clinician and relevant study team members will be informed and will follow-up with the patient directly. Clinician education will include recommended usual care activities in response to call triggers.

#### 5.4.1 Early Call Triggers (Up to 3 Months)

The research coordinator will alert the designated site clinician and appropriate study team members when a patient reports to the coordinator any of the following symptoms:

1. Persistent fevers or chills;
2. Persistent vomiting and/or inability to take medication;
3. Worsening abdominal pain despite pain medication;
4. Patient requesting discussion with medical team; and
5. Clinical concern by research coordinator (e.g., significant persistent dizziness or significant new diarrhea).

The designated site clinician and study team will be responsible for determining if further evaluation is needed and for coordinating the patient's care upon receiving notification from the research coordinator. The research coordinator will document contact with the study team by either 1) responding to the Survey Response Review report on the CODA portal if report of the call trigger was in response to a triggered surveyed question or 2) documenting by way of an internal study team call trigger tracking system concerns that are spontaneously reported.

The site RC will be responsible for initiating the Serious Adverse Event (SAE) reporting process if the reported patient concern is an SAE. See Section 9.0.

#### 5.4.2 Late Call Triggers (Beyond 3 Months)

Despite a requirement that all patients enrolled in the study have diagnostic imaging of the abdomen to exclude the possibility of a cancer, it is still possible that patients treated with antibiotics only may harbor an appendiceal mass. The research coordinator will alert the designated site clinician and study team if any of the following are reported any time after three months:

1. Complaints of signs and symptoms of recurrent appendicitis;
2. Unintentional weight loss of more than 15lbs; or
3. Diagnostic imaging consistent with appendiceal mass.

If the patient has not seen a healthcare provider to address these issues, the research coordinator will offer to connect the patient to the appropriate scheduling coordinator to make an appointment for follow-up at any of the clinical sites or the lead of the CCC.

As noted in Section 5.4.1, the research coordinator will document contacting the study team by either 1) responding to the Survey Response Review report on the CODA portal if report of the

call trigger was in response to a triggered surveyed question or 2) documenting by way of an internal study team call trigger tracking system concerns that are spontaneously reported.

Additionally, the site RC will be responsible for initiating the SAE reporting process if the reported patient concern is an SAE. See Section 9.0.

#### 5.4.3 Clinician Education

Clinicians will be advised to follow usual clinical care pathways and principles in responding to these call triggers described in Sections 5.4.1 and 5.4.2. Additionally, site clinical leads will be formally educated through training sessions on the following study materials: protocol, research coordinator call triggers, and SAE reporting process.

Clinicians will be encouraged to apply usual clinical care and potentially, repeat diagnostic imaging or colonoscopy for participants who did not have an appendectomy and have persistent gastrointestinal symptoms at or after one-year.

### 5.5 Reasons for Withdrawal

Participants may be withdrawn from the study for the following reasons:

1. Participant desires withdrawal (reasons for withdrawal will be recorded); and
2. Study investigator(s) deems it in the participant's best interest to be withdrawn (reason for withdrawal recorded).

#### 5.5.1 Handling of Withdrawals

If the participant requests to be withdrawn from the study, they will be given appropriate treatment, but will not continue with scheduled study follow-ups. If the participant withdraws from the study and withdraws consent for disclosure of future information, no further evaluations will be performed and additional data will not be collected. The investigators may retain and continue to use data collected before withdrawal of consent.

### 5.6 Compensation

#### 5.6.1 Participant Compensation

Participants will be paid up to $155 for completing the research assessments described in Table 2 (Section 5.0). Payment will be provided on a pro-rated, weighted fashion: $20 for the Baseline Assessment, $10 for the Week 1 and 2 Assessments ($20), $20 for the Week 4 Assessment, $10 for Quarterly Assessments through 12 months ($40, Months 3, 6, 9, 12), $10 for the 18-month Assessment, and $15 for the 24-month Assessment. Extended follow-up surveys at 36, 48, and 60 months will also be pro-rated at $10 per survey. Compensation will be distributed to participants centrally by UW research staff unless other arrangements have been made.

#### 5.6.2 Site and Site Lead Compensation

Each site will determine how best to allocate their funds to support this research at their hospital. Funds may be used to support study staff including site surgical and ED lead investigators, relevant clinical and research support staff, and research study supplies. Funds

for research participant compensation will not come directly from these site payments but rather provided by UW centrally as described in Section 5.6.1.

## 5.7 Retention Activities

### 5.7.1 Maintaining Contact

Participants will be asked to confirm their contact information before beginning each research assessment. They will also be asked to confirm the contact information of family members, friends, and employers who may be asked to update the participant's contact information should they be otherwise lost to follow-up. Participants may receive personalized birthday cards with $5.00 coffee-shop gift card or other small incentive annually during their birthday month.

### 5.7.2 Contact between Assessments

Three interim communications will be performed between 12 and 24 month follow-up assessments to help retain participants in the study and to improve communication with them. During the year, coordinators will:

1. Send a thank-you "mailer" to the participant in recognition of his or her participation. The mailer may include an updated site newsletter and a small token of appreciation, such as a pen with the study logo;
2. Request an update in contact information; and
3. May send a birthday card with a $5 gift card or another small incentive.

# 6.0 Outcome Assessments

## 6.1 Primary Outcome

Patient Reported Outcomes (PROs) will be assessed for adults using both generic and disease-specific surveys utilizing the QoL instruments in Table 4.

Table 4. PRO Measures.

| Validated Scale | Description |
|---|---|
| EQ-5D[20] | A preference-based instrument designed to measure generic health status across 5 dimensions of health: mobility, self-care, usual activities, pain/discomfort, and anxiety/depression, with 3 response levels (i.e., no problems, some problems, extreme problems). A Visual Analog Scale (scored 0-100) is also included. |
| 10 PROMIS Global Health Short Form[21] | A 10-item instrument measuring domains that can be scored into a Global Physical Health component and Global Mental Health component. Each item includes 5 response choices, with the exception of the common 11–point pain intensity item ("How would you rate your pain on average?" with 0=no pain and 10=worst imaginable pain). |
| Decision Regret Scale[23] | A 5-item validated questionnaire that measures regret among patients following a specific treatment decision. Scores range from 0-100 and higher scores reflect higher levels of regret. While developed for use in patients undergoing treatment for cancer, the questions are general in nature and adaptable to diverse clinical experiences. |
| GIQLI[22] | A 36-item questionnaire assessing 5 domains: GI symptoms, physical function, emotional well-being, social well-being and perception of medical treatment measured by a single item question. Each item has 5 response choices (i.e., all of the time, most of the time, some of the time, a little of the time, never). In addition, the measure produces and overall QoLscore (0-144) where higher numbers indicate greater QoL. |

## 6.2 Secondary Outcomes

### 6.2.1 Clinical Complications

Death, SAEs, and complications (reported as AEs via EMR review) will be assessed and recorded through the study surveillance period. Complications (reported as AEs) will incorporate NSQIP standards and definitions as well as events related to antibiotic-related complications.

| Serious Adverse Events | Adverse event |
|---|---|
| **Death** | |
| **Life-threatening event** | |
| Severe antibiotic reaction | Mild/moderate antibiotic reaction |
| CVA or stroke | |
| MI requiring treatment or cardiac arrest | Myocardial infarction<br>Atrial arrhythmia |
| Unplanned admit to ICU | |
| Acute Renal Failure (requiring dialysis) | Major UTI (e.g., pyelonephritis)<br>Urinary retention<br>Acute Renal Failure (no dialysis)<br>Progressive renal insufficiency |
| C. difficile colitis (requiring colon resection) | |
| Pulmonary embolism requiring therapy | DVT/PE requiring treatment |
| Coma >24 hours | |
| Septic shock (requiring pressors) | Sepsis |
| **Serious Adverse Events (continued)** | **Adverse event (continued)** |
| Bleeding requiring transfusion | |
| Newly infected prosthetic graft infection (such as a cardiac valve that gets infected or synthetic bypass graft infection secondary to appendicitis) | |
| Mechanical ventilation >48 hours | Tracheal reintubation/tracheostomy |
| Colostomy or ileostomy | NG tube replacement (non-routine)<br>Bowel obstruction<br>Ileus |
| | Organ/space infection (including peritonitis)<br>Surgical site infection<br>Intra-ab Abscess<br>Severe dehydration<br>Hernia<br>Malignant hyperthermia<br>Pneumonia<br>Other infection<br>Other post-op occurrence: unspecified<br>Other non-operative intervention: unspecified<br>Unplanned (re)operative intervention during index<br>C. difficile colitis (not requiring colon resect) |
| Other life threatening event | |
| **Rehospitalization** (other than for txt of appendicitis) | |

| | Monitored event |
|---|---|
| | Appendiceal cancer |
| | Perforation |
| | Length of stay postoperatively |

### 6.2.2 Eventual Appendectomy

Participants will be asked whether they have had an appendectomy at each contact through their four-week follow-up after the index diagnosis of appendicitis, and then surveyed at regular intervals to determine if they developed recurrent appendicitis and the resulting treatment (non-operative intervention, unplanned re-operative intervention, planned or elective appendectomy). With the participant's informed consent, the research coordinator will obtain all inpatient and outpatient medical records related to AEs or recurrent appendicitis and records will be abstracted to determine whether the AEs were associated with the incident appendicitis or whether recurrent appendicitis was confirmed. If recurrent appendicitis was confirmed, treatment and patient/clinician decision-making will be recorded on the EMR form.

Patients reporting eventual appendectomy will be surveyed to determine, from the patient perspective, the reason for eventual appendectomy.

## 7.0 Data Management and Information Security

### 7.1 Data Management

Data management will be the responsibility of the site investigators for data collected at their particular site, and the UW DCC for the combined data from all sites.

At sites, all study questionnaires completed in person or by phone (or by mail or email) will be reviewed by research coordinators for accuracy and completeness. Through the Week 2 follow-up and as needed subsequently through the 2-year follow-up, data collection is the responsibility of the study staff at the site under the supervision of the site investigator. During the study, the site investigator will maintain complete and accurate documentation for the study.

The UW DCC will provide support for data management, quality review, analysis, and reporting of the study data. The DCC will create the electronic study database using REDCap (Research Electronic Data Capture). REDCap is a secure web application for building and managing online surveys and databases.

Patient follow-up from 4-weeks on will be completed using customizable software from DatStat, Inc (Seattle, WA) to securely manage survey distribution methods, response data collection, and reporting. UW study staff will complete data audits to ensure data quality as well as accurate data-entry for surveys received by mail.

### 7.1.2 Data Quality Monitoring

The CCC and DCC will review participant enrollment, reasons for exclusion, participant demographics, and follow-up rates by site in order to assess proximity to enrollment targets, site performance, and protocol adherence.

In addition, the DCC will perform early quality assurance checks by running REDCap Data Quality reports. These reports include missing values for required fields, incorrect data

type, range checks, outliers, hidden fields that contain values, and multiple choice fields with invalid values. Values that need to be corrected will be brought to the attention of the research staff at that site.

### 7.2 Information Security

Sites:

All participant data collected on paper at sites will be stored in locked file cabinets located in the site research offices.

All data entered onto laptop/desktop computers located at sites will be secured via each site's Information Security infrastructure. Information Security at all participating sites is multi-layered and includes physical security measures, network security measures, managed servers, and desktop/laptop computers with authentication and authorization controls. All systems are designed and implemented to properly secure restricted or confidential information, including PHI, from inadvertent or unauthorized disclosure. All policies and procedures adhere to federal, state, and health system requirements. Servers, desktops, and laptops are under active management by an Integrated Technology Services Group (ITSG) at each participating site. In addition to maintaining anti-virus and firewall installations, the ITSG audits patch status of systems connected to the network and installs any missing updates or patches found. Servers have defined change control periods for operating system maintenance but are patched or updated outside the schedule as necessary to address threats. Anti-virus software is configured to update daily. The host-based firewalls restrict inbound connections to only the subnets where department workforce resides or that are needed for firewall administration. The firewall rule set on the dedicated server is further restricted to the network subnets. A specific Windows domain account is required to access computers. Domain passwords must be at least 8 characters in length, conform to complexity rules, and be changed at least every 120 days. Database systems and security policies are established and overseen by administrative staff members who do not require research funding.

RedCAP:

All data, including PHI, names, and contact information will be entered onto a HIPAA and 21 CFR Part 11- compliant database. Participant PHI, including names, contact information, health information, will be entered onto this database. Only study staff will have access to the database and each study staff member will be given separate access only after completing study-required trainings.

DatStat:

*Data Transmission and Storage*

DatStat servers, databases, and web presences employ multiple forms of security features, while security protocols are designed to protect both the data itself, as well as the participants involved in data collection efforts.

DatStat secure servers are registered with site certificates provided by AddTrust that provide for advanced encryption over the wire. As each user moves through the survey form, his/her responses are encrypted while in-transit between the browser and DatStat's server using SSL (Secure Sockets Layer) and 40, 56, or 128-bit Public Key Encryption.

All servers used for data collection are highly fault-tolerant and equipped with redundant, hot-pluggable power supplies, redundant network interfaces, and RAID 5 hot-swappable disk storage. All primary servers are plugged into a monitored, uninterruptible power supply (UPS).

*Physical security*

DatStat servers are stored in a locked server cabinet/rack, which are housed in a state-of-the-art, well-ventilated data center. Physical access to servers and data backup is restricted to a minimal number of IT professionals. The facility is secured by guards who monitor all access to the building and will notify DatStat personnel of any building compromise or intrusion.

*Logical security*

In addition to the physical safeguards listed above, the data center is monitored 24/7 by Information Technology (IT) and security staff via equipment monitoring and Closed-Circuit Television. Servers are protected from remote attacks through use of dedicated hardware firewalls (Watchguard), with auditing enabled at the recommended settings. Watchguard LiveSecurity keeps IT staff advised of all known security alerts. Firewalls closely monitor traffic to block suspicious packets from access to all systems.

Security patches are applied to DatStat servers on a timely and ongoing basis. Logs are created by the web servers to increase accountability and are essential in investigating incidents after the fact. The following are logged: failed and successful logins, attempts to access files/ directories without authority, successful and failed attempts to access sensitive data.

## 8.0 Statistical Analysis Plan and Stopping Rules

### 8.1 Randomization Overview

This is a non-inferiority based, pragmatic, randomized controlled trial (RCT) of antibiotics versus appendectomy for the treatment of uncomplicated appendicitis with a concurrent observational cohort to characterize any selection bias associated with patients choosing to participate in the randomized trial, and to support the generalizability of study results. By necessity, this is an un-blinded study (patients will know if they were randomized to appendectomy or antibiotics), and analysts will know the treatment allocation of study participants. The DCC will generate and maintain randomization lists for each practice site. We will use block randomization within practice sites, with random blocks of 4, 6, or 8 participants. Using block randomization ensures that equal numbers of participants are randomized to the intervention and control arm and that the two groups are balanced at periodic enrollment intervals. Crossover, in the conventional manner, is defined as a patient who is randomized to appendectomy who refuses to undergo surgery or someone who is randomized to antibiotics but chooses appendectomy before the antibiotics are started. For the sake of convenience in describing statistical analysis and data monitoring plans, we define the term ***early antibiotics failures*** (or *treatment changed*) as patients in the antibiotics treatment arm who were treated with appendectomy within four weeks after randomization.

### 8.2 Outcomes Measures

The primary analysis outcome is the EQ-5D at four weeks following randomization to treatment in the intention-to-treat (ITT) population. Important clinical endpoints include the following: complications or AEs associated with antibiotics or appendectomy; days until resolution of symptoms; rates of perforated appendicitis; rates of appendiceal cancer; hospital days; number of days using antibiotics beyond the initial treatment schedule; clinic visits; and caregiver/patient

"time in healthcare." Additional PRO measures include the 10 PROMIS Global Health Short Form, the Decision Regret Scale, and GIQLI.

### 8.3 General Analytic Strategy

The primary evaluation of EQ-5D at four-weeks will be conducted using an ITT analysis, where patients' data are analyzed according to the patients' randomized treatment assignment. Using this analytic approach in a non-inferiority study design comparing antibiotics treatment to appendectomy, we aim to test whether the global EQ-5D scores are not different by more than a margin of 5% (M = 5%). The margin (5%) was selected because it is within the meaningful clinical important difference (MCID) threshold of the EQ-5D, which is 5-10%.[20,24,25]

1. Primary Analysis, Aim 1 – Patient-reported QoL, as measured by the EQ-5D at four-weeks, will be assessed using a linear regression model that adjusts for an indicator of randomized treatment group assignment and for all factors used to stratify randomization (e.g., practice site). As recommended by the United States Food and Drug Administration guidelines on clinical trial design,[26] the estimated treatment effect and 97.5% one-sided confidence interval (CI) will be compared to the non-inferiority margin (M = -5%).[27-29] We will conclude that antibiotics are non-inferior to appendectomy when the entire 97.5% one-sided confidence interval is greater than M (Figure 1, example trial conclusion A). This is equivalent to a one-sided ($\alpha$=0.025) test of the null hypothesis $H_0$: $\Delta \leq$ -5%, where $\Delta$ represents the difference in mean EQ-5D at four-weeks comparing the antibiotics treatment strategy to the appendectomy treatment strategy. If the null hypothesis of $H_0$: $\Delta \leq$ -5% is rejected at the final evaluation, we will conduct a test of superiority to determine the level of statistical evidence supporting an alternative hypothesis $H_A$: $\Delta$ > 0% (Figure 1, example trial conclusion B). **For Sub Aim 1,** we will similarly evaluate non-inferiority on patient-reported QoL among the cohort *without* appendicolith.

Figure 1. Example Trial Conclusions in a Non-inferiority Trial.

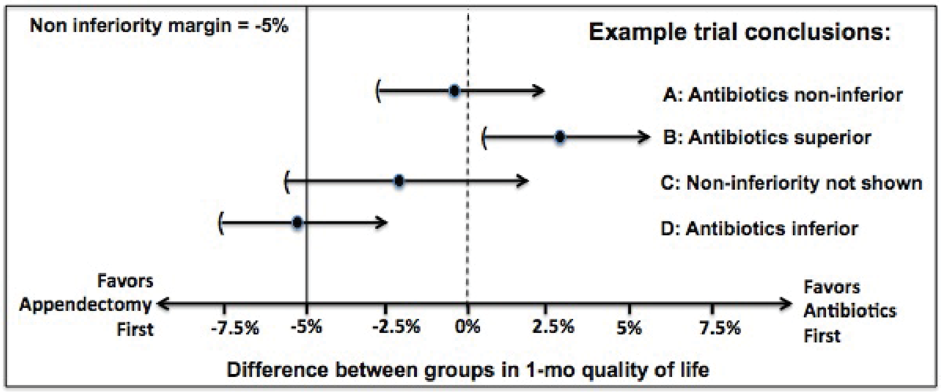


2. Secondary Analysis, Aim 1 – Secondary analyses of the primary outcome measures will include examining the entire trajectory of EQ-5D QoL measurements for each patient using linear mixed effects models for longitudinal data.[30] While patient-elected changes in treatment and early antibiotics failures are anticipated, the ITT approach is an appropriate primary analysis. We will conduct a secondary as-treated analysis of the primary outcome measure that appropriately accounts for patient- or provider-level characteristics found to be differentially represented among patients in the antibiotics

arm who are treated with appendectomy and those in the appendectomy arm who do not ultimately have surgery. Careful as-treated and secondary data analyses are of importance in understanding the effectiveness of the treatments and in assessing in which subgroups is the antibiotics treatment strategy most effective.[31] A simple as-treated analysis is problematic because randomization is broken when select patients in the antibiotics arm ultimately receive appendectomy or surgery-assigned participants who later choose to forego surgery. Thus, patients who are successfully treated with antibiotics and do not undergo appendectomy may differ demographically or clinically in ways that introduce bias in a naive as-treated analysis. Detry (JAMA 2014) recommends both an ITT and a careful as-treated analysis to address treatment changes in non-inferiority trials where non-adherence, crossover, or treatment changes are present. If we find a difference between the ITT and as-treated results, the ITT results will be considered the primary analysis.[32]

We will consider a two-stage approach for this as-treated analysis to: 1) identify subgroups that are likely to require appendectomy, therefore, should not be considered as good candidates for treatment with antibiotics, and 2) estimate the complier average causal effect (CACE), which seeks to compare the outcomes of patients treated successfully in the antibiotics arm (i.e., did not ultimately have surgery) with patients randomized to the appendectomy arm who similarly would not have crossed over **had they been randomized to the antibiotics arm**. The CACE framework considers the ITT mean in each randomized arm as being comprised of two subgroup means: 1) patients who would be successfully treated with antibiotics without appendectomy and 2) patients who would eventually require appendectomy after being treated with antibiotics. We are able to directly observe which patients in the antibiotics arm ultimately cross over, but require the CACE framework to identify similar patients in the appendectomy arm. We will use a maximum likelihood mixture modeling approach to identify the optimal comparison group from the control arm for observed compliers in the intervention arm.[33] This application of mixture modeling permits an examination of two aspects of intervention process, the prediction of early antibiotics failures in the appendectomy group and the examination the treatment effect on QoL among patients who are suitable candidates for treatment with antibiotics.[34,35]

We will also compare secondary PROs (i.e., 10 PROMIS Global Health Short Form, GIQLI, and the Decision Regret Scale) using an ITT framework and linear regression models. Similar to the primary outcome measure, secondary PROs will be assessed using model-based point estimates and associated confidence intervals. The magnitude of effect will be judged *a posteriori* using margins that are of comparable magnitude as the EQ-5D in terms of the Cohen's d statistic. In an exploratory analysis, the complete longitudinal trajectory of all PRO metrics will be assessed with linear mixed effects models.

3. Exploratory Aim A – Using only data from patients enrolled in the antibiotics arm, we will also examine the association of these characteristics with eventual appendectomy. We will employ univariate logistic regression models to identify and characterize associations with unplanned appendectomy. If we find that a number of baseline characteristics are relevant and the rate of unplanned appendectomy is high enough (e.g., >10-15%), we will use multivariable analyses to compare these characteristics among those undergoing early appendectomy (defined within the first four weeks) and eventual appendectomy (after four weeks). Due to the potentially large number of input variables, interactions, and longitudinal assessments, we will also use modern model

development methods such as the least absolute shrinkage and selection operator (LASSO) with boosting.[36,37] Boosting provides a more sophisticated approach towards building multivariable predictive models and allows nonlinear relationships between the input variables and risk of requiring appendectomy after first being treated with antibiotics. Boosting is a multiple prediction and aggregation method for classification, where a “base learner” fitting method is fitted multiple times on reweighted data and a final estimator is constructed through a linear combination of the multiple estimates. Results of these analyses will be considered hypothesis generating and may support secondary studies aimed at predictive modeling.

4. Primary Analysis, Aim 2 – Clinical endpoints (e.g., days until resolution of symptoms; rates of perforated appendicitis; extent of operation and surgical complications; complications associated with antibiotics; hospital days; number of days using antibiotics beyond the initial treatment; clinic visits; and caregiver/patient “time in healthcare”) will also be compared between ITT groups using regression models appropriate to each endpoint (e.g., linear, logistic, Poisson, or Cox proportional hazards regression models), along with a similar non-inferiority framework with margins based on MCIDs. **For Sub Aim 2** we will similarly compare clinical endpoints among the cohort *without* appendicolith.
5. Exploratory Aim B – One assumption is that patients included in the RCT are sufficiently similar to those from the general population of patients with appendicitis. Using simple univariate descriptive and comparative statistics (e.g., t-tests, Chi-squared tests), we will compare randomized patients to those in the parallel observational cohort to identify selection bias. We will also compare the primary outcome measures between randomized and non-randomized patients by examining the entire trajectory of EQ-5D QoL measurements using linear mixed effects models for longitudinal data, providing an assessment of differences in the rate of change of QoL. We will adjust for patient- or provider-level characteristics found to be differentially represented in RCT. Using methods similar to those described in the plan for Aim 1 Secondary Analyses, we will adjust for treatment received to fully assess whether patients who participate in the RCT have a different outcome trajectory than patients in the observational cohort.

### 8.4 Missing Data

We will conduct a missing data analysis to describe and characterize enrolled participants who do not provide data due to attrition. As described by Molenberghs and Kenward (2007), we will use inverse probability weighting in secondary analysis within each longitudinal regression model to inflate the weights of cases that are under-represented in the analysis due to selective attrition and/or non-participation.[38] We will also conduct sensitivity analyses using 10-fold multiple imputation to assess the robustness of the results when missing data are imputed. We will assess the sensitivity of inferences made from missing data first by using the two previously described methods for dealing with missing data and second, by imputing missing data under both pessimistic and optimistic scenarios to provide bounds on the statistical uncertainty. The characteristics of non-responders will be summarized in our final report and we will present the sensitivity of the estimated treatment effect due to alternative missing data methods.

### 8.5 Sample Size and Statistical Power

Prior studies suggest that non-clinical outcomes (e.g., loss of time from work and pain scores) were similar between groups, but did not evaluate a global QoL measure. The EQ-5D QoL

index ranges from 0 (worst QoL) to 1 (highest QoL), where anchor-based methods have shown that the MCID ranges 5%-10%. Based on data from a prior study of appendectomy with EQ-5D scores at 12 weeks, we estimate that the mean EQ-5D for appendectomy patients will be 0.90 with a standard deviation of 0.12. In order to assess QoL differences between interventions, we will recruit a total of 1552 patients assuming a 90% follow-up at four-weeks. This gives the study high power (>99%) to rule out EQ-5D differences between-groups as small as 5% (e.g., if we observe treatment differences of 0 to 2%) and 80% power if we observe a treatment difference of 3% (Table 5, below).

Previous Washington State evaluations of patients undergoing appendectomy (Table 1), identified that 2-3% of the population is African American, 10-12% Hispanic, 10-15% are Medicaid beneficiaries, and 10% will be above 64 years of age. Recruiting 1552 total patients will allow for a subgroup evaluation based on older age and Medicaid beneficiary status. Assuming 50% recruitment of these populations we anticipate including ~500 Medicaid beneficiaries and ~450 patients ≥65 years. As demonstrated in Table 5, this study also has adequate power (>80%) to rule out differences of 5% for observed treatment effect of $\Delta$=0% within subgroups as small as n=250 patients, or treatment differences of $\Delta$=-1% within subgroups of at least n=400. For this reason, we expect the study will be able to assess these two subgroups. All other subgroup evaluations will be *post hoc* and used for hypothesis development only.

Table 5. Statistical Power to Declare Non-inferiority on Patient-reported QoL, Overall and by Subgroup (Non-inferiority Margin, M = -5%, one-sided $\alpha$=0.025).

| Treatment Difference, Δ | Overall | Subgroups | | |
|---|---|---|---|---|
| | N=1552 | N=250 | N=400 | N=500 |
| -3% | 82.6% | - | - | - |
| -2% | 99.4% | - | 57.1% | 67.9% |
| -1% | 100% | 62.4% | 83.8% | 91.4% |
| 0% | 100% | 83.0% | 96.4% | 98.8% |

### 8.6 Data and Safety Monitoring Plan (DSMP)

An independent DSMB will be created to review the accruing data to: 1) ensure that study conduct, enrollment, and patient follow-up is adequate; 2) ensure that there are no serious safety concerns; and 3) assess evidence related to patient-reported QoL. The DSMB will be enlisted by the DCC in consultation with the executive committee.

The primary objective for interim analyses is to allow careful and continuing analysis of safety outcomes by randomization group. We will record and present all SAEs that occur during follow-up, but focus interim monitoring for those events that occur within the first four weeks of follow-up (since this is the time period in which the major treatment related events would be expected). The DSMB will review safety and efficacy data reports every six months and will conduct formal interim analyses for efficacy and safety once enrollment in the randomized cohort meets targets of 400 and 800-1000 participants. We anticipate these targets will align with analysis occurring at 18 and 30 months following enrollment. If recruitment in the study is slower than expected, the DSMB will be encouraged to consider as a contingency that investigators continue to recruit patients for up to 4.5 years with the goal of accomplishing the Aim 1 analysis (four-weeks) in all, acknowledging that longer term outcomes will only be assessed in a subset of patients. At sample sizes considerably smaller than that projected (Table 5), the study will have sufficient power to address Aim 1, excluding the non-inferiority margin when large differences in EQ-5D are observed (e.g., 3%).

1. Safety Monitoring – We will perform interim analyses of accruing safety data. SAEs (described in section 9.1) will be monitored throughout the course of the study. The DSMB will review tabulated SAEs by treatment group every six months; however, all deaths will be reported to the DSMB immediately. With additional guidance from the DSMB, we propose to monitor and compare three safety endpoints between randomization groups: 1) per-patient indicator of "any" SAE, 2) per-patient SAE count, and 3) all-cause mortality. The research coordinators at each practice site will monitor participants for, or be alerted by UW study staff regarding the presence of, SAEs and AEs during inpatient stays using EMR. This process includes reviews of SAEs and AEs that occur at other hospitals not included in the study, with details obtained through requested records from those hospitalizations. An adjudication process related to these events will be led by a clinician not otherwise involved in the trial. This independent safety monitor (ISM) will review SAE forms for data quality control. SAE forms needing additional specification will be returned to the site PI for completion prior to finalization. All SAEs will also be adjudicated by the surgical site lead.

   Based on data from prior studies, we anticipate that SAEs in the ITT antibiotics arm will be less common than in the appendectomy arm. Since appendectomy is considered standard of care and it is desirable to assess effectiveness within subgroups, we do not propose formal statistical stopping rules if SAE rates are lower in the antibiotics arm. However, evidence of higher SAEs in the antibiotics arm will require DSMB consideration of the trial's continuation. At the planned interim analysis, we will use one-sided tests to formally compare the event rates across the two ITT treatment groups using appropriate small sample methods such as Fisher's exact test or Poisson regression with robust standard errors for SAE per-patient counts. We will also monitor and compare the rates of SAEs in the appendectomy arm to the rate of SAEs among patients in the observational cohort who receive appendectomy to address concerns that surgery following antibiotic therapy may be associated with a higher surgical risk. For safety monitoring we will utilize a one-sided Pocock stopping boundary[38,39] ($\alpha$=0.025) where, at the discretion of the DSMB and faculty statistician, study recruitment may be discontinued if there is substantial statistical evidence for a higher safety event rate in the antibiotics arm compared to the appendectomy arm ($P<0.011$ at either interim or final analysis). In other words, the DSMB will consider stopping the trial on the basis of safety if there is substantial statistical evidence that patients randomized to the antibiotics treatment are experiencing a higher rate of SAEs than patients randomized to appendectomy.

2. Efficacy Monitoring – The DSMB will monitor patient-reported EQ-5D scores at four-weeks using a sequential monitoring boundary for futility. Conditional power will be used to assess futility, the likelihood of the trial to show non-inferiority at the final analysis. This will be done by calculating the probability of demonstrating non-inferiority in the ITT analysis, conditional on the observed results and on assumptions about the future results of the trial. Using conditional power for sequential monitoring of efficacy data, the type II error rate ($\beta$) for the study is bounded by $\beta/(1-\varepsilon)$, where $\varepsilon$ denotes the conditional power monitoring threshold. At the recommendation of Bratton et al. (2012), the DSMB will consider stopping the trial for futility (based on EQ-5D) if conditional power falls below a fixed threshold of $\varepsilon$=0.10.[40] In other words, the trial would only stop on the basis of the EQ-5D if the EQ-5D in the antibiotics arm is worse than that of patients randomized to appendectomy by a substantial margin. Futility analyses related to the EQ-5D will be balanced by the DSMB against the need to determine the rates of early

antibiotic failure overall, and in *a priori* selected subgroups (e.g., older patients and Medicaid beneficiaries), as well as other important clinical endpoints.

3. Early Antibiotics Failures Monitoring – In a non-inferiority study, treatment with appendectomy in the antibiotics arm may bias the study conclusions towards declaring non-inferiority of the antibiotics approach. In conjunction with safety and PROs, the DSMB will review the rate of eventual appendectomy in the antibiotics arm as a measure of adherence to protocol and study quality. No formal stopping rules are proposed on the basis of crossover or early antibiotics failure rates overall or within subgroups.
4. Subgroup Monitoring – As an important clinical subgroup of interest, the DSMB will actively monitor patients with appendicolith on safety, PROs, and the rate of treatment by appendectomy among those randomized to receive antibiotics. No formal stopping rules are proposed based upon results observed within the appendicolith subgroup because patient advisors determined that information for this substantial group of patients would be informative to decision makers even if only a small proportion (<25%) had successful treatment with antibiotics. Another clinical subgroup of interest includes patients treated with different antibiotic types and/or regimens and specifically those who received completely outpatient versus at least some inpatient antibiotic therapy. The DSMB will actively monitor patients based on antibiotics received and whether or not they ultimately had surgery. Subgroup monitoring will focus on safety, PROs, and the rate of eventual appendectomy. No formal stopping rules are proposed based on results observed within the antibiotic type and regimen subgroups since patient advisors determined that information for this group of patients would be informative to decision makers.
5. Revisions – These monitoring guidelines may be revised at the discretion and recommendation of the DSMB.
6. DSMB Recommendations – After each interim review of the data, the DSMB will recommend one of the following actions:
   a. Continuation of the study as is;
   b. Continuation of the study with protocol changes in the interest of safety or study accrual;
   c. Discontinue the study due to inadequate patient accrual, follow-up, or study conduct;
   d. Discontinue the study due to patient safety in the antibiotics arm; and
   e. Discontinue the study for futility, low conditional power for the primary question of interest.
6. Shell DSMB report tables are located in Appendix A.

## 9.0 Study Related SAEs

### 9.1 Identification of SAEs

Participants in the randomization and observational cohorts will be monitored for SAEs throughout their study surveillance period (enrollment through the optional extended follow-up). The occurrence of an SAE may come to the attention of study personnel during study follow-up

phone interview, by UW study staff personnel conducting follow-up, or by a study participant calling the study team or presenting for medical care. If an SAE comes to the attention of study personnel via patient or family report, the site research coordinator will be notified that an SAE form needs to be completed. The site research coordinator will then use the EMR to fill out the SAE form. The site PI will then complete the form and sign the form. The SAE form will then be submitted to the DCC who will provide it to the ISM. The ISM will review the SAE form and ask the surgical site lead for additional clarification or specification as he/she sees necessary. SAE forms for events other than death will be summarized for the DSMB every 6 months; however, deaths due to any cause will be reported to the DSMB immediately.

### 9.2 SAE Reporting

Life-threatening complications or SAEs are a very rare possibility with surgical treatment and even less likely with antibiotic treatment. SAEs are defined as one of the following conditions (also listed in section 6.2.1):

1. Death during the period of protocol-defined surveillance;
2. Life-threatening event related to the treatment or significant disability/incapacity related to the treatment; or
3. Inpatient hospitalization (other than for treatment of recurrent appendicitis).

All SAEs will be:

1. Recorded on the appropriate SAE case report form;
2. Adjudicated by the ISM;
3. Reviewed and evaluated by the DSMB;
4. Followed until satisfactory resolution or until the study investigators deem the event to be chronic or the participant to be stable;
5. Reported to the study investigators, the project managers, and site IRBs per their reporting guidelines; and
6. The study investigators will also report SAEs to PCORI in its role as funder.

### 9.3 Assignment of SAEs as Treatment Related

The determination of whether an SAE is related to the treatment will be assessed by the ISM and surgical site lead, based on supporting documentation provided by the research coordinators case reports or in consultation with the treating physician, as needed.

## 10.0 Reporting Procedures

SAEs will be captured on the appropriate source document. Information to be collected includes event description, date event occurred, date study personnel became aware of the event, investigator assessment of event severity, event outcome, and relationship to study treatment. All SAEs will also have information on action taken at time of report, date SAE resolved, outcome at time of SAE report, and, depending on the site, whether the IRB has been notified.

For SAEs, the date of resolution (or when the event is deemed stable/chronic) will be noted on the appropriate case report form.

# 11.0 Quality and Performance Management Plans

## 11.1 Quality Management Plan

A quality management plan (QMP) will be created to ensure early identification and resolution of problems or concerns related to the study. The QMP will include verification that sites have appropriately met the requirements to assure quality of all study-related activities. Each site will be responsible for maintaining accurate and complete patient charts and informed consent documentation. The QMP is in place to verify good practice. Research staff from the CCC will be responsible for the following activities in coordination with collaborating sites to verify QMP requirements are met:

1. Site Initiation Visit. This will involve review of:
    i. Regulatory approval documents;
    ii. Human subjects training documentation for all study staff;
    iii. Documentation of study-related required trainings; and
    iv. HIPAA-compliant storage plans for participant data (electronic and paper-based)
2. Site audit 6 months after study enrollment initiation. This will involve:
    i. Select chart audits;
    ii. Select audit of informed consent form documentation; and
    iii. Review of Protocol Violations and Deviations.
3. Annual Site Monitoring Visits (at a minimum) to review/complete:
    i. Regulatory approval documents;
    ii. Select chart audits;
    iii. Select audit of informed consent form documentation;
    iv. Review of Protocol Violations and Deviations. and
    v. Review of staff training logs.
4. The DCC will set up data quality checks within the data entry system which will check for appropriate and on-time data entry within relevant protocol-defined time windows, missing information, inconsistencies and logic errors. The DCC will monitor this information on a regular basis.

For sites that are found to have several inconsistencies and errors in data quality and completeness, or an increased number of protocol deviations compared to other sites, more monitoring visits will be scheduled and conducted as needed.

## 11.2 Performance Management Plan

Site study leadership teams will review enrollment rates and any outstanding needs or concerns with the CCC Director and CODA Project Manager on a regular basis. The purpose of these individual site meetings is to monitor site progress against anticipated and expected site enrollment targets. To meet study enrollment milestone targets, individual sites are expected to

screen all appendicitis patients. If screening/recruitment goals are not being met, the CCC will implement the following Performance Management Plan, consisting of:

1. Site Progress Call to review and discuss:
   i. Site specific screening and randomization rates;
   ii. Missed participants, study refusals;
   iii. Statement of work and milestones review;
   iv. Support or training needs for the site from the CCC; and
   v. Plan of action to address enrollment shortfalls
2. Interval Check-In Call:
   i. Site specific screening and randomization rates
   ii. Plan of action results

b. Follow-up Call(s) as needed:
   i. Continued training
   ii. In-person site visit / shadowing of study staff

Site performance, screening rates, is monitored on a weekly basis by the CODA Executive Committee. If a site consistently fails to meet expectations in comparison to the average study site, suspension or termination of the site subcontract may be necessary.

### 11.3 Protocol Violations and Deviations

A protocol violation is any non-compliance with the clinical trial protocol or Good Clinical Practices. The noncompliance may be either on the part of the participant, the investigator, or the study site staff and most often is related to study enrollment. Anticipated protocol violations are 1) Participant enrolled but did not meet inclusion criteria, 2) Participant enrolled but exclusion criteria present or eligibility criteria not met, 3) Consent not obtained in accordance with IRB guidelines, 4) Randomization failure, 5) Clinical team intervention outside of the protocol (other than those elected by participant), 6) Participant in antibiotics arm received antibiotics outside of protocol during hospital stay, and 7) Participant in antibiotics arm received antibiotics outside of protocol at discharge. Participants enrolled and later found to be ineligible will still be followed. Specifically, a participant enrolled into the randomization or observational cohort that is later found to not have appendicitis will still be asked to complete study assessments. To document the participant’s ineligibility, a protocol violation will be submitted. Participants enrolled in the EMR cohort who are later found not to have appendicitis will be withdrawn from the study. As a result of violations, corrective actions will be developed by the site and implemented promptly.

A protocol deviation is a smaller digression from study protocol. Deviations are monitored with consideration for protocol amendment should it become clear that the original protocol was too inflexible. The only anticipated protocol deviations are >7 hours between first administration of antibiotics and study approach by the research coordinator and not watching the video or reviewing the study pamphlet prior to study consent. Additional protocol deviations will be recorded and described.

It is the responsibility of the site to use continuous vigilance to identify and report violations and deviations within 5 working days of identification of the protocol violation/deviation.

All violations and deviations from the protocol will be addressed in study participant source documents. A completed copy of the Protocol Violations and Deviation Form will be maintained in the regulatory file, as well as in the participant's study chart. Protocol violations and deviations will be sent to the local IRB per their guidelines. The site investigator/study staff will be responsible for knowing and adhering to their IRB requirements.

## 12.0 Protection of Human Participants

We expect that most patients will have good baseline health but will not exclude patients with most stable and chronic medical conditions, other than those with complicated appendicitis and characteristics described in exclusion criteria.

Because the proposed study population will include Spanish-speaking patients, all consent documents and materials will be translated to Spanish. Women of childbearing potential will be excluded if they have a positive pregnancy test documented prior to enrollment or anticipate being pregnant in the following month. Prisoners will be excluded. There is no evidence from prior studies to suggest there will be differences in intervention effect among sex/gender or racial/ethnic subgroups.

The risks and benefits for participants enrolled in this trial are appropriately balanced. Both of the study interventions (appendectomy and IV antibiotics) are standard, and all of the drugs used in the study are currently in clinical use. Potential risks to participants include the potential AEs that are well described as complications of acute appendicitis, including appendiceal perforation, intraabdominal abscess, sepsis, pain, and loss of time from work and normal activities. Based on prior studies, it is not expected that these complications will be greater in patients assigned to the non-operative strategy group, but that is not known for certain. Patients assigned to the operative strategy will be participant to the well-described risks of surgery including adverse anesthesia reaction, perforation of viscus, wound infection, or bleeding. These risks would be similar to those that patients with AUA would encounter as part of standard care. All patients will also have potential risks of adverse reaction to antibiotics including allergic reaction or antibiotic-associated and *Clostridium difficile* colitis. Other risks include potential loss of patient confidentiality through the collection of study data. It is expected that the benefits of the study (including avoidance of surgery and its associated risks in the non-operative group and the benefits of the knowledge gained that will improve patient choices in the future) justify the potential risks.

The alternative to participation in the clinical trial is receipt of usual care, which currently most often involves urgent surgical appendectomy with peri-operative antibiotics. Patients will be informed of alternatives to participation in the informed consent process.

Informed consent is a process that is initiated prior to the individual's agreeing to participate in the study and continuing throughout the individual's study participation. Extensive discussion of risks and possible benefits of both treatment options will be provided to the participants and their families using standardized materials including a web-based video. Consent forms describing in detail the study interventions, study procedures, and risks are given to the participant and documentation of informed consent will be required prior to initiating treatment assignment if patient consents to randomization.

Consent forms will be IRB-approved and the participant will be given sufficient time to read and review the document and discuss with their family member, friend, or legal representative. If they require assistance to reach their family member, friend, or legal representative, such as the use of a telephone, that will be facilitated. After this, they will be specifically asked if they have any questions or concerns, which will be addressed, or would like more time to consider their

participation, which will also be provided. The study investigator or coordinator will explain the research study to the participant and answer any questions that may arise. The participants will sign the informed consent prior to completing any study related activities, including randomization if the participant were to choose that participation option. The participants may withdraw consent at any time throughout the course of the trial. A copy of the informed consent will be given to the participants for their records. The rights and welfare of the participants will be protected by emphasizing to them that they will still be able to receive medical care at the facility if they decline to participate in this study.

Participant confidentiality is strictly held in trust by the participating investigators and their staff.

## 13.0 Ancillary Studies

An ancillary study subcommittee within the CCC will work with interested investigators, representatives of PCORI and CODA stakeholders to coordinate, seek funding for, facilitate and plan ancillary studies that may be linked to the CODA trial.

## 14.0 References

1. Chang DC, Shiozawa A, Nguyen LL, et al. Cost of inpatient care and its association with hospital competition. J Am Coll Surg 2011;212:12-9.
2. Sauerland S, Jaschinski T, Neugebauer EA. Laparoscopic versus open surgery for suspected appendicitis. Cochrane Database Syst Rev 2010;(10):CD001546. doi:CD001546.
3. Coldrey E. Treatment of acute appendicitis. Br Med J 1956;2:1458-61.
4. Antibiotics resurface as alternative to removing appendix 2015. Kolata G. (Accessed Nov. 11, 2015, at http://www.nytimes.com/2015/05/19/health/antibiotics-resurface-as-alternative-to-removing-appendix.html?_r=0).
5. Wojciechowicz KH, Hoffkamp HJ, van Hulst RA. Conservative treatment of acute appendicitis: an overview. Int Marit Health 2010;62:265-72.
6. Styrud J, Eriksson S, Nilsson I, et al. Appendectomy versus antibiotic treatment in acute appendicitis. a prospective multicenter randomized controlled trial. World J Surg 2006;30:1033-7.
7. Hansson J, Korner U, Khorram-Manesh A, Solberg A, Lundholm K. Randomized clinical trial of antibiotic therapy versus appendicectomy as primary treatment of acute appendicitis in unselected patients. Br J Surg 2009;96:473-81.
8. Eriksson S, Granstrom L. Randomized controlled trial of appendicectomy versus antibiotic therapy for acute appendicitis. Br J Surg 1995;82:166-9.
9. Malik AA, Bari SU. Conservative management of acute appendicitis. J Gastrointest Surg 2009;13:966-70.
10. Vons C, Barry C, Maitre S, et al. Amoxicillin plus clavulanic acid versus appendicectomy for treatment of acute uncomplicated appendicitis: an open-label, non-inferiority, randomised controlled trial. Lancet 2011;377:1573-9.
11. Salminen P, Paajanen H, Rautio T, et al. Antibiotic therapy vs appendectomy for treatment of uncomplicated acute appendicitis: the APPAC randomized clinical trial. JAMA 2015;313:2340-8.
12. Mason RJ, Moazzez A, Sohn H, Katkhouda N. Meta-analysis of randomized trials comparing antibiotic therapy with appendectomy for acute uncomplicated (no abscess or phlegmon) appendicitis. Surg Infect (Larchmt) 2012;13:74-84.
13. McCutcheon BA, Chang DC, Marcus LP, et al. Long-term outcomes of patients with nonsurgically managed uncomplicated appendicitis. J Am Coll Surg 2014;218:905-13.

14. Carr NJ. The pathology of acute appendicitis. Ann Diagnost Pathol 2000;4:46-58.
15. Mason RJ. Surgery for appendicitis: is it necessary? Surg Infect (Larchmt) 2008;9:481-8.
16. Teixeira PG, Sivrikoz E, Inaba K, Talving P, Lam L, Demetriades D. Appendectomy timing: waiting until the next morning increases the risk of surgical site infections. Ann Surg 2012;256:538-43.
17. Eko FN, Ryb GE, Drager L, Goldwater E, Wu JJ, Counihan TC. Ideal timing of surgery for acute uncomplicated appendicitis. N Am J Med Sci 2013;5:22-7.
18. Swidsinski A, Dorffel Y, Loening-Baucke V, et al. Acute appendicitis is characterised by local invasion with Fusobacterium nucleatum/necrophorum. Gut 2011;60:34-40.
19. Solomkin JS, Mazuski JE, Bradley JS, et al. Diagnosis and management of complicated intra-abdominal infection in adults and children: guidelines by the Surgical Infection Society and the Infectious Diseases Society of America. Surg Infect (Larchmt) 2010;11:79-109.
20. Rabin R, de Charro F. EQ-5D: a measure of health status from the EuroQol Group. Ann Med 2001;33:337-43.
21. Amtmann D, Cook KF, Johnson KL, Cella D. The PROMIS initiative: involvement of rehabilitation stakeholders in development and examples of applications in rehabilitation research. Arch Phys Med Rehabil 2011;92:S12-9.
22. Eypasch E, Williams JI, Wood-Dauphinee S, et al. Gastrointestinal quality of life index: development, validation and application of a new instrument. Br J Surg 1995;82:216-22.
23. Brehaut JC, O'Connor AM, Wood TJ, et al. Validation of a decision regret scale. Med Decis Making 2003;23:281-92.
24. Le QA, Doctor JN, Zoellner LA, Feeny NC. Minimal clinically important differences for the EQ-5D and QWB-SA in post-traumatic stress Disorder (PTSD): results from a doubly randomized preference trial (DRPT). Health Qual Life Outcomes 2013;11:59,7525-11-59.
25. Koumarelas K, Theodoropoulos GE, Spyropoulos BG, Bramis K, Manouras A, Zografos G. A prospective longitudinal evaluation and affecting factors of health related quality of life after appendectomy. Int J Surg 2014;12:848-57.
26. Anonymous ICH Harmonised Tripartite Guideline. Statistical principles for clinical trials. International Conference on Harmonisation E9 Expert Working Group. Stat Med 1999;18:1905-42.
27. Turner L, Shamseer L, Altman DG, et al. Consolidated standards of reporting trials (CONSORT) and the completeness of reporting of randomised controlled trials (RCTs) published in medical journals. Cochrane Database Syst Rev 2012;11:MR000030.
28. Piaggio G, Elbourne DR, Altman DG, Pocock SJ, Evans SJ, CONSORT Group. Reporting of noninferiority and equivalence randomized trials: an extension of the CONSORT statement. JAMA 2006;295:1152-60.
29. Calvert M, Blazeby J, Altman DG, et al. Reporting of patient-reported outcomes in randomized trials: the CONSORT PRO extension. JAMA 2013;309:814-22.
30. Diggle PJ, Heagerty PJ, Liang KY, Zeger SL. Analysis of longitudinal data. London: Oxford University Press, 2002.
31. Sitlani CM, Heagerty PJ, Blood EA, Tosteson TD. Longitudinal structural mixed models for the analysis of surgical trials with noncompliance. Stat Med 2012;31:1738-60.
32. Detry MA, Lewis RJ. The intention-to-treat principle: how to assess the true effect of choosing a medical treatment. JAMA 2014;312:85-6.
33. Angrist J, Imbens G. Two-stage least squares estimation of average causal effects in models with variable treatment intensity. J Amer Statist Assoc 1995;90:431-42.
34. Little R, Yau L. Statistical techniques for analyzing data from prevention trials: Treatment of no-shows using Rubin’s causal model. Psychol Methods 1998;3:147-59.

35. Bloom H. Accounting for no-shows in experimental evaluation designs. Evaluation Review 1984;8:225-46.
36. Tibshirani R. Regression shrinkage and selection via the lasso. J Royal Statistics Society B 1996;58:267-88.
37. Hastie T, Tibshirani R, Friedman J. The elements of statistical learning. New York: Springer, 2009.
38. Molenberghs G, Kenward MG. Missing data in clinical studies. J Trop Pediatr 2007;53:294.
39. Pocock SJ. Group sequential methods in the design and analysis of clinical trials. Biometrika 1977;64:191-9.
40. Bratton DJ, Williams HC, Kahan BC, Phillips PP, Nunn AJ. When inferiority meets non-inferiority: implications for interim analyses. Clin Trials 2012;9:605-9.
