# Supplementary material for: Association of Patient Belief About Success of Antibiotics for Appendicitis and Outcomes: A Secondary Analysis of the CODA Randomized Clinical Trial
Source: JAMA Surg. 2022 Oct 5;157(12):1080–7. doi: 10.1001/jamasurg.2022.4765 (PMC9535504; doi:10.1001/jamasurg.2022.4765)
Supplement: Supplement 2. — Statistical Analysis Plan [file jamasurg-e224765-s002.pdf]

Note: Changes between first and final plans are noted immediately below, followed by the original SAP and final SAP

Summary of Changes to the CODA Statistical Analysis Plan

Research Aims

- Sub Aim 1 modified to include patients with appendicolith in addition to without appendicolith to reflect the protocol.
- Exploratory Aim A modified to reflect time windows of interest.
- Sub Aim 2 modified to include patients with appendicolith in addition to without appendicolith to reflect the protocol.

Study Design and Treatment Allocation

- Block sizes changed from 4, 6, 8 and 10 to 4, 6, and 8.
- Definition of "early antibiotic failure" removed.

Outcome Measures:

- Additional outcome measures of interest, defined in the protocol, were added.

General Analytic Strategy: Unchanged.

Primary Analysis, Aim 1:

- Plan for Sub Aim 1 modified to include patients with appendicolith in addition to without appendicolith to reflect the protocol.
- A plan for patients who were randomized using the incorrect appendicolith status was added.

Aim 1 Secondary Analyses

- GEE added as an option for analyzing longitudinal data.
- Per protocol analysis added.

Exploratory Aim A Analysis Plan:

- Plan for defining appendectomies by reason and time added.

Aim 2 Analysis Plan

- Specific clinical endpoints removed since described previously.
- Plan for Sub Aim 2 modified to include patients with appendicolith in addition to without appendicolith to reflect the protocol.

Exploratory Aim B Analysis Plan:

- GEE added as an option for analyzing longitudinal data.

Additional Exploratory Subgroups:

- Added in most recent version to reflect the protocol.

Original plan starts here:

Research Aims

Aim 1: Compare PROs in patients randomized to antibiotics-first or appendectomy.

Sub Aim 1. Compare patient reported outcomes (PROs) in patients **without appendicolith** randomized to the antibiotics or appendectomy strategy.

Exploratory Aim A. Assess the rate of eventual appendectomy after starting the antibiotics treatment regimens in the first week, early (1-4 weeks) and late (2-24 months) periods and identify patient clinical characteristics (e.g., appendicolith) as well as clinician and practice site characteristics associated with eventual appendectomy in the antibiotic therapy group.

.

Aim 2: Compare clinical outcomes in patients randomized to antibiotics-first or appendectomy.

Sub Aim 2. Compare clinical outcomes in patients **without appendicolith** randomized to the antibiotics or appendectomy strategy.

Exploratory Aim B: Compare randomized patients to those in a concurrent observational cohort to identify selection characteristics and outcome differences between the two groups.

Study Design and Treatment Allocation: This is a non-inferiority based, pragmatic, randomized controlled trial of antibiotics-first vs. appendectomy for the treatment of uncomplicated appendicitis with a concurrent observational cohort to characterize any selection bias associated with patients choosing to participate in the randomized trial, and to support the generalizability of study results. By necessity, this is an un-blinded study (patients will know if they were randomized to appendectomy or antibiotics), and analysts will know the treatment allocation of study participants. The DCC will generate and maintain randomization lists for each practice site. We will use block randomization within practice sites, with random blocks of 4, 6, 8 or 10 subjects. Using block randomization ensures that equal numbers of subjects are randomized to the intervention and control arm and that the two groups are balanced at periodic enrollment intervals. We will also stratify randomization on race to ensure balanced treatment assignments across racial subgroups. Crossover, in the conventional manner, is defined as a patient who is randomized to appendectomy who refuses to undergo surgery or someone who is randomized to antibiotics but chooses appendectomy before the antibiotics are started. For the sake of convenience in describing statistical analysis and data monitoring plans, we define the term ***early antibiotics failures*** (or *treatment changed*) as patients in the antibiotics treatment arm who were treated with appendectomy with 4 weeks after randomization.

Outcome Measures: The primary outcome is the EQ-5D at 4 weeks following randomization to treatment. Secondary patient reported outcome measures include the 10 PROMIS Global Health Short Form, the Decision Regret Scale, and Gastrointestinal Quality of Life Index (GIQLI). Clinical endpoints include: days until resolution of symptoms, rates of perforated appendicitis, extent of operative and surgical complications, complications associated with antibiotics, hospital days, number of days using antibiotics beyond the initial treatment schedule, clinic visits, and caregiver/patient "time in healthcare".

General Analytic Strategy: The primary evaluation of EQ-5D at 4 weeks will be conducted using an intention-to-treat (ITT) analysis, where patients' data are analyzed according to the patients' randomized treatment assignment. Using this analytic approach in a non-inferiority study design comparing antibiotics-first treatment to appendectomy, we aim to test whether the global EQ-5D scores are not different by more than a margin of 5% (M = 5%). The margin (5%) was selected because it is within the meaningful clinical important difference (MCID) threshold of the EQ-5D, which is 5-10%[1-3].

Primary Analysis, Aim 1: Patient-reported QoL, as measured by the EQ-5D at 4 weeks, will be assessed using a linear regression model that adjusts for an indicator of randomized treatment group assignment and for all factors used to stratify randomization (e.g., practice site, race). As recommended by the United States Food

and Drug Administration guidelines on clinical trial design[4], the estimated treatment effect and 97.5% one-sided confidence interval (CI) will be compared to the non-inferiority margin (M = -5%)[5-7]. We will conclude that antibiotics are non-inferior to appendectomy when the entire 97.5% one-sided confidence interval is greater than M, as in example scenario A (Figure 1). This is equivalent to a one-sided (alpha=0.025) test of the null hypothesis H_0: $\Delta \leq -5\%$, where Δ represents the difference in mean EQ-5D at 4-weeks comparing antibiotics-first to appendectomy-first. If the null hypothesis of H_0: $\Delta \leq -5\%$ is rejected at the final evaluation, we will then conduct a test of superiority to determine the level of statistical evidence supporting an alternative hypothesis H_A: $\Delta > 0\%$ (i.e., scenario B of Figure 1). **For Sub Aim 1,** we will similarly evaluate non-inferiority on patient-reported QoL among the cohort *without* appendicolith.

Figure 1. Example trial conclusions in a non-inferiority trial.

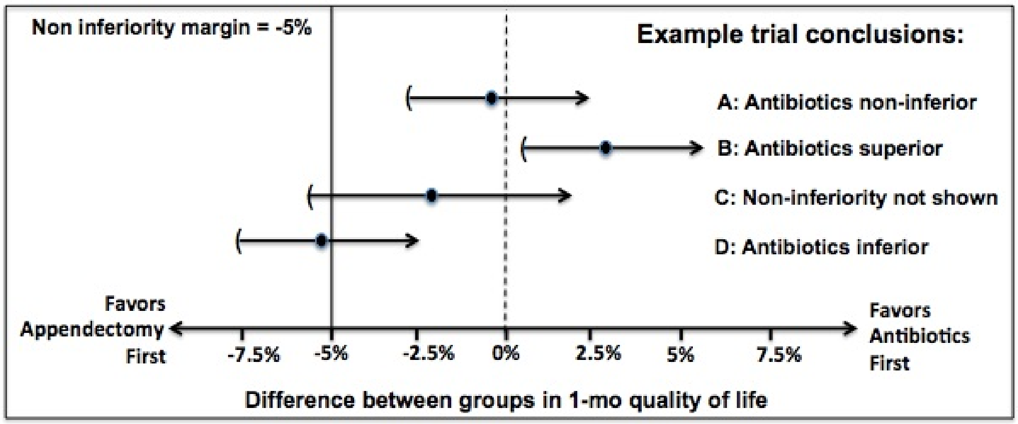

<u>**Aim 1 Secondary Analyses**</u>: Secondary analyses of the primary outcome measures will include examining the entire trajectory of EQ-5D QoL measurements for each patient using linear mixed effects models for longitudinal data.[8] While crossovers and early antibiotics failures are anticipated, the ITT approach is an appropriate primary analysis. We will conduct a secondary as-treated analysis of the primary outcome measure that appropriately accounts for patient- or provider-level characteristics found to be differentially represented among patients who crossover and patients in the antibiotics arm who are treated with appendectomy. Careful as-treated and secondary data analyses are of elevated importance towards understanding the effectiveness of the treatments and in assessing for whom antibiotics-first is an appropriate treatment.[9] A simple as-treated analysis is problematic because randomization is broken when select patients in the antibiotics-first arm ultimately receive appendectomy. Thus, patients who are successfully treated with antibiotics and do not undergo appendectomy may differ demographically or clinically in ways that introduce bias in a naive as-treated analysis. Detry (JAMA 2014) recommends both an ITT and a careful as-treated analysis to address treatment changes in non-inferiority trials where non-adherence, crossover, or treatment changes are present. If we find a difference between the ITT and as-treated results, the ITT results will be considered the primary analysis.[10]

We will consider a two-stage approach for this as-treated analysis: 1) To identify subgroups that are likely to require appendectomy and therefore should not be considered as good candidates for treatment with antibiotics-first, and 2) To estimate the complier average causal effect (CACE), which seeks to compare the outcomes of patients treated successfully in the antibiotics-first arm (i.e., did not ultimately have surgery) with patients randomized to the appendectomy arm who similarly would not have crossed over **had they been randomized to the antibiotics-first arm**. The CACE framework considers the ITT mean in each randomized arm as being comprised of two subgroup means: patients who would be successfully treated with antibiotics-first without appendectomy, and patients who would eventually require appendectomy after being treated with antibiotics-first. We are able to directly observe which patients in the antibiotics-first arm ultimately cross over, but require the CACE framework to identify similar patients in the appendectomy arm. We will use a maximum likelihood mixture modeling approach to identify the optimal comparison group from the control arm for observed compliers in the intervention arm.[11] This application of mixture modeling permits an examination of two aspects of intervention process, the prediction of early antibiotics failures in the appendectomy-first group and the examination the treatment effect on quality of life among patients who are suitable candidates for treatment with antibiotics first.[12]

<u>**Analysis of Secondary Patient-Reported Outcomes**</u>: We will also compare secondary patient reported

outcomes (i.e., 10 PROMIS Global Health Short Form, GIQLI, and the Decision Regret Scale) using an ITT framework and linear regression models. Similar to the primary outcome measure, secondary patient-reported outcomes will be assessed using model-based point estimates and associated confidence intervals. The magnitude of effect will be judged a posteriori using margins that are of comparable magnitude as the EQ-5D in terms of the Cohen's d statistic. In an exploratory analysis, the complete longitudinal trajectory of all PRO metrics will be assessed with linear mixed effects models.

Exploratory Aim A Analysis Plan: Using only data from patients enrolled in the antibiotics-first arm, we will also examine the association of these characteristics with eventual appendectomy. We will employ univariate logistic regression models to identify and characterize associations with unplanned appendectomy. If we find that a number of baseline characteristics are relevant and the rate of unplanned appendectomy is high enough (e.g., >10-15%), we will use multivariable analyses to compare these characteristics among those undergoing early appendectomy (defined within the first 4 weeks) and eventual appendectomy (4 weeks to 2 years). Due to the potentially large number of input variables, interactions, and longitudinal assessments we will also use modern model development methods such as the LASSO with boosting.[13,14] Boosting provides a more sophisticated approach towards building multivariable predictive models and allows nonlinear relationships between the input variables and risk of requiring appendectomy after first being treated with antibiotics. Boosting is a multiple prediction and aggregation method for classification, where a "base learner" fitting method is fitted multiple times on reweighted data and a final estimator is constructed through a linear combination of the multiple estimates. Results of these analyses will be considered hypothesis generating and may support secondary studies aimed at predictive modeling.

Aim 2 Analysis Plan: Clinical endpoints (e.g., days until resolution of symptoms, rates of perforated appendicitis, extent of operation and surgical complications, complications associated with antibiotics, hospital days, number of days using antibiotics beyond the initial treatment, clinic visits, and caregiver/patient "time in healthcare") will also be compared between ITT groups using regression models appropriate to each endpoint (e.g., linear, logistic, Poisson, or Cox proportional hazards regression models), along with a similar non-inferiority framework with margins based on MCIDs. **For Sub Aim 2,** we will similarly evaluate clinical endpoints among the cohort *without* appendicolith.

Exploratory Aim B Analysis Plan: One assumption is that patients included in the randomized trial are sufficiently similar to those from the general population of patients with appendicitis. Using simple univariate descriptive and comparative statistics (e.g., t-tests, Chi-squared tests), we will compare randomized patients to those in the parallel observational cohort to identify selection bias. We will also compare the primary outcome measures between randomized and non-randomized patients by examining the entire trajectory of EQ-5D QoL measurements using linear mixed effects models for longitudinal data, providing an assessment of differences in the rate of change of quality of life. We will adjust for patient- or provider-level characteristics found to be differentially represented in RCT. Using methods similar to those described in the plan for Aim 1 Secondary Analyses, we will adjust for treatment received to fully assess whether patients who participate in the RCT have a different outcome trajectory than patients in the observational cohort.

Missing Data: We will conduct a missing data analysis to describe and characterize enrolled participants who do not provide data due to attrition. As described by Molenberghs and Kenward (2007), we will use inverse probability weighting in secondary analysis within each longitudinal regression model to inflate the weights of cases that are under-represented in the analysis due to selective attrition and/or non-participation. We will also conduct sensitivity analyses using 10-fold multiple imputation to assess the robustness of the results when missing data are imputed. We will assess the sensitivity of inferences made from missing data first by using the two previously described methods for dealing with missing data and second, by imputing missing data under both pessimistic and optimistic scenarios to provide bounds on the statistical uncertainty. The characteristics of non-responders will be summarized in our final report and we will present the sensitivity of the estimated treatment effect due to alternative missing data methods.

Statistical Analysis Plan References

1. Rabin R, de Charro F. EQ-5D: a measure of health status from the EuroQol Group. Ann Med. 2001 Jul;33(5):337-43. PMID:11491192

2. Le QA, Doctor JN, Zoellner LA, Feeny NC. Minimal clinically important differences for the EQ-5D and QWB-SA in Post- traumatic Stress Disorder (PTSD): results from a Doubly Randomized Preference Trial (DRPT). Health Qual Life Outcomes. 2013 Apr 12;11:59. PMID:23587015

3. Koumarelas K, Theodoropoulos GE, Spyropoulos BG, et al. A prospective longitudinal evaluation and affecting factors of health related quality of life after appendectomy. Int J Surg. 2014;12(8):848-57. PMID:25019228

4. International Conference on Harmonisation E9 Expert Working Group. (1999). ICH harmonised tripartite guideline: statistical principles for clinical trials. Stat Med 18(15):1905-42. PMID:10532877

5. Turner L, Shamseer L, Altman DG, et al. Consolidated standards of reporting trials (CONSORT) and the completeness of reporting of randomised controlled trials (RCTs) published in medical journals. Cochrane Database Syst Rev. 2012 Nov 14;11:MR000030. PMID:23152285

6. Piaggio G, Elbourne DR, Pocock SJ, Evans SJ, Altman DG; CONSORT Group. Reporting of noninferiority and equivalence randomized trials: extension of the CONSORT 2010 statement. JAMA. 2012 Dec 26;308(24):2594-604. PMID:23268518

7. Calvert M, Blazeby J, Altman DG, Revicki DA, Moher D, Brundage MD; CONSORT PRO Group. Reporting of patient-reported outcomes in randomized trials: the CONSORT PRO extension. JAMA. 2013 Feb 27;309(8):814-22. PMID:23443445

8. Diggle PJ, Heagerty PJ, Liang KY, Zeger SL. Analysis of Longitudinal Data. Second Edition ed: Oxford University Press; 2002.

9. Sitlani CM, Heagerty PJ, Blood EA, Tosteson TD. Longitudinal structural mixed models for the analysis of surgical trials with noncompliance. Stat Med. 2012 Jul 20;31(16):1738-60. PMID:22344923. PMCID:PMC3876882

10. Detry MA, Lewis RJ. The intention-to-treat principle: how to assess the true effect of choosing a medical treatment. JAMA. 2014 Jul 2;312(1):85-6. PMID:25058221

11. Angrist J, Imbens G. Two-stage least squares estimation of average causal effects in models with variable treatment intensity. Journal of the American Statistical Association. 1995;90:431–442.

12. Little R, Yau L. Statistical techniques for analyzing data from prevention trials: Treatment of no-shows using Rubin's causal model. Psychological Methods. 1998;3:147–159; Bloom, H. (1984). Accounting for No-Shows in Experimental Evaluation Designs. Evaluation Review 8(2), 225-246.

13. Tibshirani, R. (1996). Regression shrinkage and selection via the lasso, *J. Royal Statistics Society B.* **58**: 267-288.

14. Hastie T, Tibshirani R, Friedman J. The Elements of Statistical Learning. Second Edition: Springer; 2009.

15. Molenberghs G, Kenward MG. Missing Data in Clinical Studies. J Trop Pediatr 2007;53(4):294. Retrieved at: http://tropej.oxfordjournals.org/content/53/4/294.full

16. Pocock SJ (1977). "Group sequential methods in the design and analysis of clinical trials". Biometrika 64 (2): 191–9. doi:10.1093/biomet/64.2.191.

17. Bratton DJ, Williams HC, Kahan BC, Phillips PP, Nunn AJ. When inferiority meets non-inferiority: implications for interim analyses. Clin Trials. 2012 Oct;9(5):605-9. doi: 10.1177/1740774512453220. Epub 2012 Jul 13.

Final SAP starts here:

Research Aims

Aim 1: Compare PROs in patients randomized to antibiotics-first or appendectomy.

Sub Aim 1. Compare patient reported outcomes (PROs) in patients with and without appendicolith randomized to the antibiotics or appendectomy strategy.

Exploratory Aim A. Assess the rate of eventual appendectomy after starting the antibiotics treatment regimens in the first 48 hours, early (30 days) and late (1 year) periods and identify patient clinical characteristics (e.g., appendicolith) as well as clinician and practice site characteristics associated with eventual appendectomy in the antibiotic therapy group.

Aim 2: Compare clinical outcomes in patients randomized to antibiotics-first or appendectomy.

Sub Aim 2. Compare clinical outcomes in patients with and without appendicolith randomized to the antibiotics or appendectomy strategy.

Exploratory Aim B: Compare randomized patients to those in a concurrent observational cohort to identify selection characteristics and outcome differences between the two groups.

Study Design and Treatment Allocation: This is a non-inferiority based, pragmatic, randomized controlled trial of antibiotics-first vs. appendectomy for the treatment of uncomplicated appendicitis with a concurrent observational cohort to characterize any selection bias associated with patients choosing to participate in the randomized trial, and to support the generalizability of study results. By necessity, this is an un-blinded study (patients will know if they were randomized to appendectomy or antibiotics), and analysts will know the treatment allocation of study participants. The DCC will generate and maintain randomization lists for each practice site. We will use block randomization within practice sites, with random blocks of 4, 6, or 8 subjects. Using block randomization ensures that equal numbers of subjects are randomized to theintervention and control arm and thatthe two groups are balanced at periodic enrollment intervals. We will also stratify randomization on presence appendicolith determined by imaging to ensure balanced treatmentassignments across this important subgroup. Crossover, in the conventional manner, is defined as a patient who is randomized to appendectomy who refuses to undergo surgery or someone who is randomized to antibiotics but chooses appendectomy before or after the antibiotics are started. Please see below for our specific definition of protocol compliance that will be used for a per-protocol analysis.

Outcome Measures: The primary outcome is the EQ-5Dat 4 weeks following randomization to treatment. Secondary patient reported outcome measures include the 10 PROMIS Global Health Short Form, the Decision Regret Scale, Gastrointestinal Quality of Life Index (GIQLI), healthcare utilization, days missed of work, days a caregiver missed of work, and work productivity. Clinical endpoints include: resolution of symptoms, rates of appendectomy, rates of appendicitis-related events (e.g., perforated appendicitis, additional course of antibiotics, more extensive surgical procedure, and drainage procedures), extent of operative and surgical complications, neoplasm and other pathology findings, adverse and serious adverse events, hospital days, clinic visits, and caregiver/patient “time in healthcare”.

General Analytic Strategy: The primary evaluation of EQ-5Dat 4 weeks will be conductedusing an intention-to-treat (ITT)analysis, where patient data areanalyzed according to the patient’s randomized treatment assignment. Using this analytic approach in a non-inferiority study design comparing antibiotics-first treatment to appendectomy, we aim to test whether the global EQ-5D scores are not different by more than a margin of 5% (M = 5%). The margin (5%) was selected because it is within the meaningful clinical important difference (MCID) threshold of the EQ-5D, which is 5-10%[1-3].

<u>Primary Analysis. Aim 1:</u> Patient-reported QoL, as measured by the EQ-5D at 4 weeks, will be assessed using a linear regression model that adjusts for an indicator of randomized treatment group assignment and for all factors used to stratify randomization (i.e., practice site and appendicolith). As recommended by the United States Food and Drug Administration guidelines on clinical trial design[4], the estimated treatment effect and 97.5% one-sided confidence interval (CI) will be compared to the non-inferiority margin (M = -5%)[5-7]. We will conclude that antibiotics are non-inferior to appendectomy when the entire 97.5% one-sided confidence interval is greater than M, as in example scenario A (Figure 1). This is equivalent to a one-sided (alpha=0.025) test of the null hypothesis H_0: $\Delta \leq -5\%$, where Δ represents the difference in mean EQ-5D at 4-weeks comparing antibiotics-first to appendectomy-first. If the null hypothesis of H_0: $\Delta \leq -5\%$ is rejected at the final evaluation, we will then conduct a test of superiority to determine the level of statistical evidence supporting an alternative hypothesis H_A: $\Delta > 0\%$ (i.e., scenario B of Figure 1). **For Sub Aim 1,** we will similarly evaluate non-inferiority on patient-reported QoL among the cohort *with and without* appendicolith. Patients whose randomization was performed for the incorrect appendicolith group (e.g., research coordinator selected the wrong button during randomization) will be assigned to the group representing their true status.

Figure 1. Example trial conclusions in a non-inferiority trial.

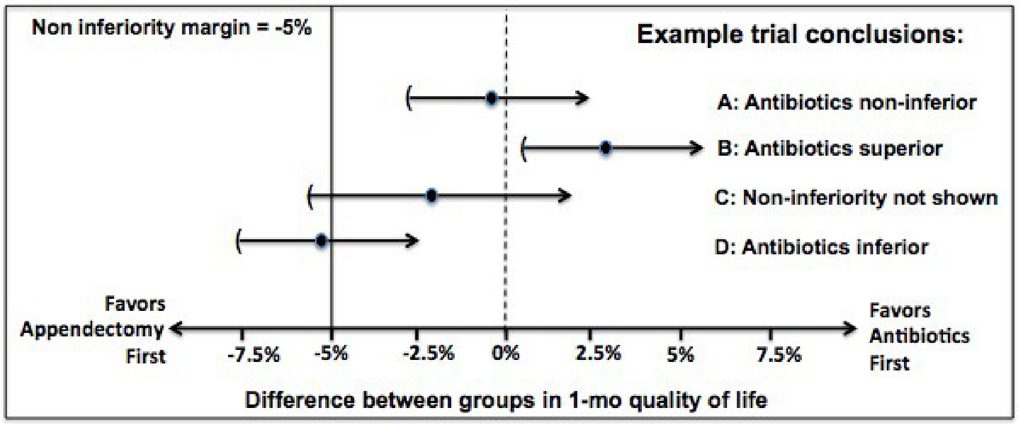

<u>Aim 1 Secondary Analyses</u>: Secondary analyses of the primary outcome measures will include examining the entire trajectory of EQ-5D QoL measurements for each patient using statistical methods for longitudinal data such as linear mixed effects models or generalized estimating equations (GEE).[8]

While crossovers are anticipated, the ITT approach is an appropriate primary analysis. We will conduct a secondary as-treated analysis of the primary outcome measure that appropriately accounts for patient- or provider-level characteristics found to be differentially represented among patients who crossover and patients in the antibiotics arm who are treated with appendectomy. Careful as-treated and secondary data analyses are of elevated importance towards understanding the effectiveness of the treatments and in assessing for whom antibiotics-first is an appropriate treatment.[9] A simple as-treated analysis is problematic because randomization is broken when select patients in the antibiotics-first arm ultimately receive appendectomy. Thus, patients who are successfully treated with antibiotics and do not undergo appendectomy may differ demographically or clinically in ways that introduce bias in a naive as-treated analysis. Detry (JAMA 2014) recommends both an ITT and a careful per-protocol analysis to address treatment changes in non-inferiority trials where non-adherence, crossover, or treatment changes are present. If we find a difference between the ITT and as-treated results, the ITT results will be considered the primary analysis.[10]

Per-Protocol Analysis: It is important to consider subjects who do not follow the protocol since these individuals bias treatment group comparisons toward no difference, but this is the alternative hypothesis rather than the null hypothesis for non-inferiority trials. Therefore, it is recommended that an ITT analysis is augmented with a per-protocol analysis that considers only those subjects from both treatment groups who adhered to the protocol. Although such a subset analysis may have bias due to patient factors associated

with non-adherence to the protocol it is a valuable companion analysis that does not suffer from non-adherence bias in the way that ITT analysis may. Our protocol states the conditions that are considered to adhere to the protocol, therefore we use the following criteria:

- All patients: exclude not randomized in accordance to the protocol (i.e., meets all inclusion/exclusion criteria, randomization assignment followed)
- Surgical group: exclude subjects whose randomization assignment was ignored.
- Antibiotics group: exclude subjects whose randomization assignments were ignored, were not given protocol-described antibiotics, or who underwent an appendectomy (for non-protocol indications, as described by sites).

For the antibiotics group we have a direct measurement of whether eventual surgery is compliant with the protocol as reported by the sites. In the per-protocol analysis we will exclude subjects according to the criterion above, and then analyze EQ5D using mean differences and the associated confidence interval to judge non-inferiority similar to our ITT analysis plan.

As-Treated Analysis: We will consider a two-stage approach for this as-treated analysis: 1) To identify subgroups that are likely to require appendectomy and therefore should not be considered as good candidates for treatment with antibiotics-first, and 2) To estimate the complier average causal effect (CACE), which seeks to compare the outcomes of patients treated successfully in the antibiotics-first arm (i.e., did not ultimately have surgery) with patients randomized to the appendectomy arm who similarly would not have crossed over **had they been randomized to the antibiotics-first arm**. The CACE framework considers the ITT mean in each randomized arm as being comprised of two subgroup means: patients who would be successfully treated with antibiotics-first without appendectomy, and patients who would eventually require appendectomy after being treated with antibiotics-first. We are able to directly observe which patients in the antibiotics-first arm ultimately cross over, but require the CACE framework to identify similar patients in the appendectomy arm. We will use a maximum likelihood mixture modeling approach to identify the optimal comparison group from the control arm for observed compliers in the intervention arm.[11] This application of mixture modeling permits an examination of two aspects of intervention process, the prediction of early antibiotics failures in the appendectomy-first group and the examination the treatment effect on quality of life among patients who are suitable candidates for treatment with antibiotics first.[12]

Analysis of Secondary Patient-Reported Outcomes: We will also compare secondary patient reported outcomes (i.e., 10 PROMIS Global Health Short Form, GIQLI, and the Decision Regret Scale) using an ITT framework and linear regression models. Similar to the primary outcome measure, secondary patient-reported outcomes will be assessed using model-based point estimates and associated confidence intervals. The magnitude of effect will be judged a posteriori using margins that are of comparable magnitude as the EQ-5D in terms of the Cohen's d statistic. In an exploratory analysis, the complete longitudinal trajectory of all PRO metrics will be assessed with linear mixed effects models.

Exploratory Aim A Analysis Plan: Using only data from patients enrolled in the antibiotics-first arm, we will also examine the association of these characteristics with eventual appendectomy. The primary analysis will consider appendectomies performed for any reason. A secondary analysis will define appendectomies as those performed for: (1) acute clinical reason 0-30 days post randomization, (2) non-acute clinical reason and (3) recurrence after a period of symptom resolution (30+ days post randomization).

We will employ univariate logistic regression models to identify and characterize associations with unplanned appendectomy. If we find that a number of baseline characteristics are relevant and the rate of unplanned appendectomy is high enough (e.g., >10-15%), we will use multivariable analyses to compare these characteristics among those undergoing early appendectomy (defined within the first 30 days) and eventual appendectomy (4 weeks to 2 years). Due to the potentially large number of input variables, interactions, and longitudinal assessments we will also use modern model development methods such as the LASSO with boosting.[13,14] Boosting provides a more sophisticated approach towards building multivariable

predictive models and allows nonlinear relationships between the input variables and risk of requiring appendectomy after first being treated with antibiotics. Boosting is a multiple prediction and aggregation method for classification, where a "base learner" fitting method is fitted multiple times on reweighted data and a final estimator is constructed through a linear combination of the multiple estimates. Results of these analyses will be considered hypothesis generating and may support secondary studies aimed at predictive modeling.

Aim 2 Analysis Plan: Clinical endpoints (see Outcome Measures above) will also be compared between ITT groups using regression models appropriate to each endpoint (e.g., linear, logistic, Poisson, or Cox proportional hazards regression models), along with a similar non-inferiority framework with margins based on MCIDs when available. **For Sub Aim 2,** we will similarly evaluate clinical endpoints among the cohort with and without appendicolith. The goal is to estimate treatment effects within each subgroup based on appendicolith.

Exploratory Aim B Analysis Plan: One assumption is that patients included in the randomized trial are sufficiently similar to those from the general population of patients with appendicitis. Using simple univariate descriptive and comparative statistics (e.g., t-tests, Chi-squared tests), we will compare randomized patients to those in the parallel observational cohort to identify selection bias. We will also compare the primary outcome measures between randomized and non-randomized patients by examining the entire trajectory of EQ-5D QoL measurements using linear mixed effects models or GEE for longitudinal data, providing an assessment of differences in the rate of change of quality of life. We will adjust for patient- or provider-level characteristics found to be differentially represented in RCT. Using methods similar to those described in the plan for Aim 1 Secondary Analyses, we will adjust for treatment received to fully assess whether patients who participate in the RCT have a different outcome trajectory than patients in the observational cohort.

Missing Data: We will conduct a missing data analysis to describe andcharacterize enrolled participants who do not provide data due to attrition. Specifically, we use 10-fold multiple imputation to assess the robustness of the results when missing data are imputed. In addition, as described by Molenberghs and Kenward (2007), we will also use inverse probability weighting in secondary analysis within each longitudinal regression model to inflate the weights of cases that are under-represented in the analysis due to selective attrition and/or non-participation. We will assess the sensitivity of inferences made from missing data first by using the two previously described methods for dealing with missing data and second, by imputing missing data under both pessimistic and optimistic scenarios to provide bounds on the statistical uncertainty. The characteristics of non-responders will be summarized in our final report and we will present the sensitivity of the estimated treatment effect due to alternative missing data methods.

Additional Exploratory Subgroups: We will also evaluate outcomes in selection subgroups including advanced age, sex, comorbid conditions and insurance status. For these analyses we will focus on evaluation of statistical interactions with treatment to assess treatment effect heterogeneity.

Statistical Analysis Plan References

1. Rabin R, de Charro F. EQ-5D: a measure of health status from the EuroQol Group. Ann Med. 2001 Jul;33(5):337-43. PMID:11491192

2. Le QA, Doctor JN, Zoellner LA, Feeny NC. Minimal clinically important differences for the EQ-5D and QWB-SA in Post- traumatic Stress Disorder (PTSD): results from a Doubly Randomized Preference Trial (DRPT). Health Qual Life Outcomes. 2013 Apr 12;11:59. PMID:23587015

3. Koumarelas K, Theodoropoulos GE, Spyropoulos BG, et al. A prospective longitudinal evaluation and affecting factors of health related quality of life after appendectomy. Int J Surg. 2014;12(8):848-57. PMID:25019228

4. International Conference on Harmonisation E9 Expert Working Group. (1999). ICH harmonised tripartite guideline: statistical principles for clinical trials. Stat Med 18(15):1905-42. PMID:10532877

5. Turner L, Shamseer L, Altman DG, et al. Consolidated standards of reporting trials (CONSORT) and the completeness of reporting of randomised controlled trials (RCTs) published in medical journals. Cochrane Database Syst Rev. 2012 Nov 14;11:MR000030. PMID:23152285

6. Piaggio G, Elbourne DR, Pocock SJ, Evans SJ, Altman DG; CONSORT Group. Reporting of noninferiority and equivalence randomized trials: extension of the CONSORT 2010 statement. JAMA. 2012 Dec 26;308(24):2594-604. PMID:23268518

7. Calvert M, Blazeby J, Altman DG, Revicki DA, Moher D, Brundage MD; CONSORT PRO Group. Reporting of patient-reported outcomes in randomized trials: the CONSORT PRO extension. JAMA. 2013 Feb 27;309(8):814-22. PMID:23443445

8. Diggle PJ, Heagerty PJ, Liang KY, Zeger SL. Analysis of Longitudinal Data. Second Edition ed: Oxford University Press; 2002.

9. Sitlani CM, Heagerty PJ, Blood EA, Tosteson TD. Longitudinal structural mixed models for the analysis of surgical trials with noncompliance. Stat Med. 2012 Jul 20;31(16):1738-60. PMID:22344923. PMCID:PMC3876882

10. Detry MA, Lewis RJ. The intention-to-treat principle: how to assess the true effect of choosing a medical treatment. JAMA. 2014 Jul 2;312(1):85-6. PMID:25058221

11. Angrist J, Imbens G. Two-stage least squares estimation of average causal effects in models with variable treatment intensity. Journal of the American Statistical Association. 1995;90:431–442.

12. Little R, Yau L. Statistical techniques for analyzing data from prevention trials: Treatment of no-shows using Rubin's causal model. Psychological Methods. 1998;3:147–159; Bloom, H. (1984). Accounting for No- Shows in Experimental Evaluation Designs. Evaluation Review 8(2), 225-246.

13. Tibshirani, R. (1996). Regression shrinkage and selection via the lasso, *J. Royal Statistics Society B.* **58**: 267-288.

14. Hastie T, Tibshirani R, Friedman J. The Elements of Statistical Learning. Second Edition: Springer; 2009.

15. Molenberghs G, Kenward MG. Missing Data in Clinical Studies. J Trop Pediatr 2007;53(4):294. Retrieved at: http://tropej.oxfordjournals.org/content/53/4/294.full

16. Pocock SJ (1977). "Group sequential methods in the design and analysis of

clinical trials". Biometrika 64 (2): 191–9. doi:10.1093/biomet/64.2.191.

17. Bratton DJ, Williams HC, Kahan BC, Phillips PP, Nunn AJ. When inferiority meets non-inferiority: implications for interim analyses. Clin Trials. 2012 Oct;9(5):605-9. doi: 10.1177/1740774512453220. Epub

2012 Jul 13.
