# Supplementary material for: Association of Patient Belief About Success of Antibiotics for Appendicitis and Outcomes: A Secondary Analysis of the CODA Randomized Clinical Trial
Source: JAMA Surg. 2022 Oct 5;157(12):1080–7. doi: 10.1001/jamasurg.2022.4765 (PMC9535504; doi:10.1001/jamasurg.2022.4765)
Supplement: Supplement 3. — eFigure. Distribution of Beliefs in CODA Antibiotics Group Appendix 1. Description of Variables Appendix 2. Description of Multiple Imputation Appendix 3. Description of Propensity Scores Appendix 4. Complete Case Data Appendix 5. The CODA Trial Sites and Site Leads [file jamasurg-e224765-s003.pdf]

Supplementary Online Content

Writing Group for the CODA Collaborative. Association of patient belief about success of antibiotics for appendicitis and outcomes: a secondary analysis of the CODA randomized clinical trial. *JAMA Surg*. Published online October 5, 2022.
doi:10.1001/jamasurg.2022.4765

eFigure. Distribution of Beliefs in CODA Antibiotics Group

Appendix 1. Description of Variables

Appendix 2. Description of Multiple Imputation

Appendix 3. Description of Propensity Scores

Appendix 4. Complete Case Data

Appendix 5. The CODA Trial Sites and Site Leads

This supplementary material has been provided by the authors to give readers additional information about their work.

© 2022 Writing Group for the CODA Collaborative. *JAMA Surgery.*

eFigure. Distribution of Beliefs in CODA Antibiotics Group

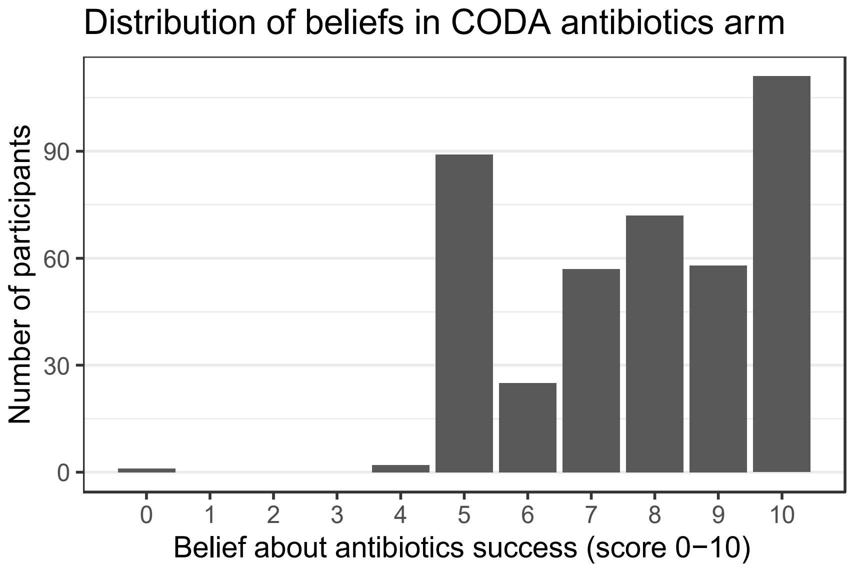

Histogram of raw belief scores (0 = unsuccessful, 5 = unsure, 10 = completely successful) in the cohort that was randomized to antibiotics and answered the belief question before learning their treatment assignment (n=425). Ten participants were excluded from this figure for missing data.

© 2022 Writing Group for the CODA Collaborative. *JAMA Surgery.*

Appendix 1. Description of Variables

Age: Participant age at baseline, in years. Obtained from participant self-report. This was treated as a continuous measure.

Sex: Participant sex at baseline (male or female). Obtained from participant self-report, supplemented by electronic medical records (EMR) when missing.

BMI: Body mass index was calculated from participant height and weight. Weight was measured at baseline, and height was taken at baseline or obtained from records within 1 year of enrollment. This was treated as a continuous measure. Data on BMI were missing for 133/425 participants.

Race: Participant race at baseline was obtained from self-report and supplemented by the EMR when missing. Due to how uncommon they were in this subgroup, people who selected American Indian or Alaska Native, Native Hawaiian or Pacific Islander, and people who identified as multiple races were combined with people who selected 'Other' for race, and this diverse group is referred to as 'Other/multiple.' Data on race were missing for 4/425 participants.

Hispanic ethnicity: Whether a participant identified as Hispanic (yes/no) at baseline was obtained from self-report and supplemented by EMR when missing.

Health literacy help: On the baseline survey, participants were asked "How often do you need to have someone help you when you read instructions, pamphlets, or other written material from your doctor or pharmacy?" with possible answers always, often, sometimes, rarely, or never. Due to sparseness in some categories, responses were grouped into never/rarely and sometimes/often/always. Data on health literacy help were missing for 15/425 participants.

Education: On the baseline survey, participants were asked, "What is the highest education level you have attained?" and were given the following options to choose from: less than high school; high school graduate or GED; some college, no degree; associate degree or academic program; occupational/technical/vocational program; Bachelor's degree; Master's degree (e.g., MA, MS, MEng, MEd, MBA); professional school degree (e.g., MD, DDS, DVM, JD); and Doctoral degree (e.g., PhD, EdD). Due to sparseness in many categories, responses were grouped into "high school/GED or less" (less than high school, high school graduate, or GED) and "some beyond high school/GED" (all other options). Data on education were missing for 4/425 participants.

© 2022 Writing Group for the CODA Collaborative. *JAMA Surgery.*

Below poverty or Medicaid/state program: This composite measure combines several questions gathered at index. Participants were asked about their household income and size, and these answers were used to determine whether the participant's household income was below the federal poverty line. Participants were also asked "What is the primary source of your health care coverage?" which included an option for "Medicaid or other state program." Participants are counted as 'yes' for the composite poverty/Medicaid variable if they either have a household income below the federal poverty level or use Medicaid or another state program. Participants are counted as 'no' for poverty/Medicaid if they meet neither of those criteria. Data on poverty/Medicaid were missing for 93/425 participants.

Charlson: The modified Charlson comorbidity score at baseline was calculated from information provided by the participant and the EMR. This was treated as a continuous measure. Data on the Charlson score were missing for 2/425 participants.

Alvarado score: The Alvarado score at baseline was calculated from information provided by the participant and the EMR. This was treated as a continuous measure. Data on the Alvarado score were missing for 24/425 participants.

WBC count: White blood cell count (in 1000/microL units) was abstracted from participants' charts during their index health care encounter by trained research staff. This was treated as a continuous measure. Data on WBC count were missing for 2/425 participants.

Duration of symptoms: The number of days with symptoms at baseline was reported to the nearest half day and abstracted from participants' charts by trained research staff. Data on duration of symptoms were missing for 1/425 participants.

Average pain previous 7 days: Participants were asked at baseline, "In the past 7 days, how would you rate your pain on average?" from 0 ("No pain") to 10 ("Worst imaginable pain"). This was treated as a continuous measure. Data on average pain were missing for 10/425 participants.

Fever: History of fever at baseline was determined by participants' subjective report of a history of fever recorded in the chart and abstracted by trained research staff.

Appendiceal diameter: Appendiceal diameter was obtained from computed tomography (CT) or ultrasound performed prior to participant enrollment. This was treated as a continuous measure. Data on appendiceal diameter were missing for 56/425 participants.

© 2022 Writing Group for the CODA Collaborative. *JAMA Surgery.*

Appendicolith: Whether a participant had an appendicolith at baseline was obtained from CT or ultrasound performed prior to participant enrollment.

© 2022 Writing Group for the CODA Collaborative. *JAMA Surgery.*

Appendix 2. Description of Multiple Imputation

In addition to belief, outcomes, and baseline factors used in the propensity score, other participant-reported and medical record-based variables were included in the imputation process (see list below). Estimates were pooled across the 20 imputation sets using Rubin's rules and are shown with corresponding 95% confidence intervals.

Variables included in the multivariate imputation by chained equations (MICE) algorithm, in addition to the three outcomes, are all baseline factors from the propensity scores (age, sex, body mass index [BMI], average pain in the previous 7 days, appendiceal diameter, appendicolith, health literacy help, education, poverty/Medicaid, race, Hispanic ethnicity), and belief:

Duration of symptoms (<1 day vs. 1+ days); white blood cell (WBC) count; fever; any nausea, vomiting, or anorexia (yes vs. no); any perforation, abscess, or moderate or severe peri-appendiceal fat stranding on imaging (yes vs. no); whether participants were worried about hospital bills (yes vs. no); PROMIS instrumental support score; Alvarado score; height (cm); weight (kg); number of adults who bring income into the household; employment (employed vs. student vs. unemployed/retired/other); frequency of physical activity at work (most or all of the time vs. some of the time or less vs. not employed); site of enrollment; symptom resolution at 2 weeks.

All variables were measured at baseline unless indicated otherwise.

© 2022 Writing Group for the CODA Collaborative. *JAMA Surgery.*

Appendix 3. Description of Propensity Scores

Propensity scores for the intermediate and completely successful belief groups were calculated using predicted probabilities from multinomial logistic regression. Variables included in the propensity score were selected among baseline factors available in the CODA trial that could plausibly confound the relationship between beliefs and outcomes: age (continuous), sex (male or female), body mass index (BMI, continuous), race (White, Black or African American, Asian, or Other/Multiple), Hispanic ethnicity (yes or no), health literacy help (sometimes or more vs. never or rarely), education (high school/GED or less vs. some beyond high school/GED), below poverty or Medicaid/state program (yes or no), average pain in the previous 7 days (continuous), appendiceal diameter (continuous), and appendicolith (yes or no). Based on observations from previous analyses, for BMI and appendiceal diameter a quadratic term was also included in the propensity score model. In the model for the adjusted risk differences (aRDs), propensity scores were included using a natural spline framework with two knots each, located at quantiles. Because of its importance in prior CODA analyses, appendicolith was included as both a factor in the propensity scores and an additional adjustment variable in the model for the aRDs.[1]

1. Writing Group for the CODA Collaborative, Monsell SE, Voldal EC, et al. Patient Factors Associated With Appendectomy Within 30 Days of Initiating Antibiotic Treatment for Appendicitis. JAMA Surg. 2022;157(3):e216900.

© 2022 Writing Group for the CODA Collaborative. *JAMA Surgery.*

Appendix 4. Complete Case Data

Outcome		Overall (n=425)	Unsuccessful/unsure (n=92)	Intermediate (n=212)	Completely successful (n=111)
Appendectomy within 30 days	Yes	80 (20%)	24 (27%)	39 (19%)	15 (14%)
	No	330 (80%)	64 (73%)	168 (81%)	90 (86%)
Persistent signs or symptoms up to 30 days	Yes	126 (33%)	36 (46%)	55 (28%)	31 (32%)
	No	258 (67%)	43 (54%)	143 (72%)	67 (68%)
High regret or dissatisfaction at 30 days	Yes	63 (17%)	15 (19%)	35 (18%)	13 (14%)
	No	312 (83%)	62 (81%)	160 (82%)	81 (86%)

For the three outcomes of interest in this analysis, the table shows counts and percentages both overall and by belief group using only complete case data. The 10 patients missing information on belief are included in the overall column only

© 2022 Writing Group for the CODA Collaborative. *JAMA Surgery.*

Appendix 5. The CODA Trial Sites and Site Leads

Sites and Site Leads: Bellevue Hospital Center New York University School of Medicine**:** Patricia Ayoung-Chee, MD, MPH, William Chiang, MD; Beth Israel Deaconess Medical Center**:** Charles Parsons, MD, Stephen R. Odom, MD, Nathan I. Shapiro, MD, MPH; Boston University Medical Center**:** Sabrina E. Sanchez, MD, MPH, F. Thurston Drake, MD, MPH; Columbia University Medical Center**:** Katherine Fischkoff, MD, Aleksandr Tichter, MD; Harbor-University of California Los Angeles Medical Center: Daniel A. DeUgarte, MD, Amy H. Kaji, MD, PhD; Harborview Medical Center**:** Heather Evans, MD, MS, Joseph Cuschieri, MD, Amber K. Sabbatini, MD, MPH; Henry Ford Health Hospital**:** Jeffrey Johnson, MD, Joe H. Patton, MD; Madigan Army Medical Center**:** Vance Sohn, MD, Karen McGrane, MD; Maine Medical Center**:** Damien W. Carter, MD; The Ohio State University Wexner Medical Center**:** Steven Steinberg, MD, David Evans, MD; Olive View-University of California Los Angeles Medical Center**:** Darin Saltzman MD, PhD, David A. Talan, MD, Gregory J. Moran, MD; Providence Regional Medical Center Everett**:** Careen S. Foster, MD, Brandon Tudor, MD; Rush University Medical Center**:** Thea P. Price, MD; Swedish Medical Center**:** Katherine A. Mandell, MD, MPH; Tisch Hospital New York University Langone Medical Center**:** Patricia Ayoung-Chee, MD, MPH, William Chiang, MD; UCHealth University of Colorado Hospital**:** Lisa Ferrigno, MD, MPH, Matthew Salzberg, MD, MBA; University of Iowa Hospitals and Clinics**:** Dionne A. Skeete, MD, Brett A. Faine, PharmD, MS; University of Michigan Medical Center**:** Pauline K. Park, MD, Hasan B. Alam, MD; University of Mississippi Medical Center: Matthew E. Kutcher, MD, MS, Alan Jones, MD; McGovern Medical School at The University of Texas Health Science Center at Houston (UTHealth)**:** Lillian S. Kao, MD, MS; University of Texas Lyndon B. Johnson General Hospital**:** Mike K. Liang, MD; University of Washington Medical Center**:** Giana H. Davidson, MD, MPH, Amber K. Sabbatini, MD, MPH; Vanderbilt University Medical Center**:** Callie M. Thompson, MD, Wesley H. Self, MD, MPH; Virginia Mason Medical Center**:** Abigail Wiebusch, MD, Juliana T. Yu, MD; Weill Cornell Medical Center**:** Robert J. Winchell, MD, Sunday Clark, ScD, MPH.

Site abbreviations: *BID* = Beth Israel Deaconess Medical Center, BMC = Boston University Medical Center, COL = Columbia University Medical Center, *HFH* = Henry Ford Health System, HMC = Harborview Medical Center-UW Medicine, IOW = University of Iowa Hospitals & Clinics, LBJ = University of Texas Lyndon B. Johnson General Hospital, *MAD* = Madigan Army Medical Center (affiliated with University of Washington) *MIS* = University of Mississippi Medical Center, *MMC* = Maine Medical Center, *NYB* = Bellevue Hospital Center NYU School of Medicine, NYT = Tisch Hospital NYU Langone Medical Center, *OSU* = Ohio State University Medical Center, PRE = Providence Regional Medical Center Everett, RUSH = Rush University Medical Center, SWE = Swedish Medical Center, *UCD* = UCHealth University of Colorado Hospital, Denver, UCH = Harbor UCLA Medical Center, UCO = Olive View-UCLA Medical Center, *UOM* = University of Michigan Medical Center, UTH = McGovern Medical School at The University of Texas Health Science Center at Houston (UTHealth), UOW = University of Washington Medical Center-UW Medicine, VAN = Vanderbilt University Medical Center, VM = Virginia Mason Medical Center, WMC = Weill Cornell Medical Center

© 2022 Writing Group for the CODA Collaborative. *JAMA Surgery.*

Collaborators: Collaborators: BID: Charles S.Parsons, MD; Nathan I. Shapiro, MD, MPH; Stephen R.Odom, MD; BMC: F. Thurston Drake, MD, MPH; COL: Aleksandr Tichter, MD; Randall Cooper; HFH: Jeffrey Johnsson, MD; Joe H.Patton, MD; Lillian Adrianna Hayes; Alyssa Hayward; HMC:Erika Wolff, PhD; Farhood Farjah, MD, MPH; Hikmatullah Arif; Joseph Cuschieri, MD; Kelsey Pullar, MPH; Laura Hennessey, RN; IOW: Cathy Fairfield, BSN; Dionne Skeete, MD; LBJ: Debbie Lew, MPH; Karla Bernardi, MD; Naila Dhanani, MD; Oscar Olavarria, MD; Stephanie Marquez; Tien C. Ko, MD; MAD: Karen McGrane, MD; Vance Sohn, MD; MIS: Deepti Patki, MS; Matthew Kutcher, MD; Rebekah K. Peacock, RN; MMC: Bruce Chung, MD; Damien Carter, MD; David MacKenzie, MD; Debra Burris, RN; Joseph Mack, MD; Terilee Gerry, MS, RN; NYB: Mohamad Abouzeid, MD; Paresh Shah, MD; Prashant Sinha, MD; NYT: Jason Maggi, MD; Kristyn Pierce, MS; Marcovalerio Melis, MD; Patricia Ayoung-Chee, MD, MPH; OSU: Amy Rushing, MD; Bruce Wolfe, MD; JonWisler, MD; Steven Steinberg, MD; PRE: Brandon Tudor, MD; Careen Foster, MD; Shaina Schaetzel, MD; SWE: Dayna Morgan, MSNBC; John Tschirhart, MD; Julie Wallick, BS, BA; Katherine Mandell, MD, MPH; Ryan Martinez, MD; Sean Wells, MD; UCD: LisaFerrigno, MD, MPH; UCH: Brant Putnam, MD; Dennis Kim, MD; Erin C. Howell, MD; Lara H. Spence, MD; Ross Fleischman, MD; UCO: Darin Saltzman, MD, PhD; Debbie Mireles, NP; Formosa Chen, MD, MPH; Gregory J.Moran, MD; Kavitha Pathmarajah, MPH; Lisandra Uribe, BA; MelindaGibbons, MD; Paul J Schmit, MD; Robert Bennion, MD; UOM: Cindy Hsu, MD, PHD; Hasan B.Alam, MD; Nathan Haas, MD; Norman Olbrich; Pauline Park, MD; UOW: Amber Sabbatini, MD, MPH; Daniel Kim, MD; Danielle C. Lavallee, PhD; Estell J.Williams, MD; Karen Horvath, MD; Zoe Parr, MD; VAN: Karen F. Miller, RN, MPA; Kelly M. Moser; VMM:Abigail Wiebusch, MD; Julianna Yu, MD; Scott Osborn, MD; WCM: Billie Johnsson, MS; Karla Ballman, PhD; Lauren Mount, MD; Robert J. Winchell, MD; Sunday Clark, ScD, MPH

Executive Committee: Bonnie Bizzell, MBA, MEd (Chair, Patient Advisory Board); Bryan Comstock, MS (Operations Director, Data Coordinating Center); Giana Davidson, MD, MPH (Chair, Clinical Coordinating Center); Erin Fannon (Senior Project Manager); David R. Flum, MD, MPH (Co-Principal Investigator); Patrick J. Heagerty, PhD, MS (Director, Data Coordinating Center); Larry G. Kessler, ScD (Chair, Executive Committee); Anusha Krishnadasan, PhD (Project Manager, California); Danielle C. Lavallee, PharmD, PhD (Director, Stakeholder Coordinating Center); Sarah O. Lawrence (Director, Stakeholder Coordinating Center); Sarah E, Monsell, MS (Lead Biostatistician); Kelsey Pullar, MPH (Research Coordinator Lead); David A. Talan, MD (Co-Principal Investigator); Erika Wolff, PhD (Executive Director, SORCE (UOW))

Patient Advisory Board: Meridith Weiss, Kimberly Deeney, Heather VanDusen, Elliott Skopin, Mary Guiden, Miriam Hernandez

National Advisory Board: Emily E. Anderson, PhD, MPH; Darrell A. Campbell, Jr., MD; Fergal Fleming, MD; David B. Hoyt, MD; J.J. Tepas III, MD (Deceased); Richard W. Whitten, MD; SreyRam Kuy, MD; Daniel S. Lessler, MD, MHA.

Data Safety and Monitoring Board: Karla Ballman, PhD; Thomas Diflo, MD; Bruce Wolfe, MD; Arden Morris, MD; Donald Yealy, MD. Patient Advisors: Kathleen O’Connor, EdD; Olga Owens, N-PC

© 2022 Writing Group for the CODA Collaborative. *JAMA Surgery.*
