# Supplementary material for: Association of Patient Belief About Success of Antibiotics for Appendicitis and Outcomes: A Secondary Analysis of the CODA Randomized Clinical Trial
Source: JAMA Surg. 2022 Oct 5;157(12):1080–7. doi: 10.1001/jamasurg.2022.4765 (PMC9535504; doi:10.1001/jamasurg.2022.4765)
Supplement: Supplement 4. — Nonauthor Collaborators [file jamasurg-e224765-s004.pdf]

Supplemental Online Content: Nonauthor Collaborators
*First name, last name, and suffix (if applicable) are required and will appear in PubMed.

*Group Name(s): CODA Collaborative						
***First Name and Middle Initial(s)**	***Last Name**	Academic Degrees	Institution	Location (city, state/province, country)	Role or Contribution, eg, chair, principal investigator	Group (if more than 1 Group listed in the byline) and/or Subgroup (eg, Steering Committee)
Mohamad	Abouzeid	MD	Tisch Hospital NYU Langone Medical Center	New York, NY, USA	Co-Investigator	Clinical Coordinating Center
Hasan B.	Alam	MD	University of Michigan Medical Center	Ann Arbor, MI, USA	Co-Investigator	Clinical Coordinating Center
Hikmatullah	Arif		Harborview Medical Center	Seattle, WA, USA	Study Staff	Clinical Coordinating Center
Karla	Ballman	PhD	Weill Cornell Medical Center	New York, NY, USA	Clinical Reviewer	Data Safety and Monitoring Board
Robert	Bennion	MD	Olive View- University of California Los Angeles Medical Center	Los Angeles, CA, USA	Co-Investigator	Clinical Coordinating Center
Karla	Bernardi	MD	University of Texas Lyndon B. Johnson General Hospital	Houston, TX, USA	Co-Investigator	Clinical Coordinating Center
Debra	Burris	RN	Maine Medical Center	Portland, ME, USA	Study Staff	Clinical Coordinating Center
Damien	Carter	MD	Maine Medical Center	Portland, ME, USA	Co-Investigator	Clinical Coordinating Center
Patricia	Chee	MD, MPH	NYT	New York, NY, USA	Co-Investigator	Clinical Coordinating Center
Formosa	Chen	MD, MPH	Olive View- University of California Los Angeles Medical Center	Los Angeles, CA, USA	Co-Investigator	Clinical Coordinating Center
Bruce	Chung	MD	Oregon Health & Science University	Portland, OR, USA	Co-Investigator	Clinical Coordinating Center
Sunday	Clark	ScD, MPH	Weill Cornell Medical Center	New York, NY, USA	Co-Investigator	Clinical Coordinating Center
Randall	Cooper		Columbia University Medical Center	New York, NY, USA	Co-Investigator	Clinical Coordinating Center
Joseph	Cuschieri	MD	HMC	Seattle, WA, USA	Co-Investigator	Clinical Coordinating Center
Kimberly	Deeney	BA	PAB	Seattle, WA, USA	Advisor	Patient Advisory Board
Naila	Dhanani	MD	University of Texas Lyndon B. Johnson General Hospital	Houston, TX, USA	Co-Investigator	Clinical Coordinating Center
Thomas	Diflo	MD	Westchester Medical Center	Valhalla, NY, USA	Clinical Reviewer	Data Safety and Monitoring Board
F. Thurston	Drake	MD, MPH	Boston Medical Center	Boston, MA, USA	Co-Investigator	Clinical Coordinating Center
Cathy	Fairfield	BSN	University of Iowa Healthcare	Iowa City, IA, USA	Study Staff	Clinical Coordinating Center
Farhood	Farjah	MD, MPH	Harborview Medical Center	Seattle, WA, USA	Co-Investigator	Clinical Coordinating Center
Lisa	Ferrigno	MD, MPH	UCD	Denver, CO, USA	Co-Investigator	Clinical Coordinating Center
Katherine	Fischkoff	MD, MPH	Swedish Medical Center	Seattle, WA, USA	Co-Investigator	Clinical Coordinating Center
Ross	Fleischman	MD	Harbor- University of California Los Angeles Medical Center	Los Angeles, CA, USA	Co-Investigator	Clinical Coordinating Center
Careen	Foster	MD	Providence Regional Medical Center Everett	Everett, WA, USA	Co-Investigator	Clinical Coordinating Center
Terilee	Gerry	MS, RN	Maine Medical Center	Portland, ME, USA	Study Staff	Clinical Coordinating Center
Melinda	Gibbons	MD	UCO	Symar, CA, USA	Co-Investigator	Clinical Coordinating Center
Mary	Guiden	BA	PAB	Fort Collins, CO, USA	Advisor	Patient Advisory Board
Nathan	Haas	MD	University of Michigan Medical Center	Ann Arbor, MI, USA	Co-Investigator	Clinical Coordinating Center
Lillian Adrianna	Hayes		Henry Ford Health Hospital	Detroit, MI, USA	Study Staff	Clinical Coordinating Center
Alyssa	Hayward		Henry Ford Health Hospital	Detroit, MI, USA	Study Staff	Clinical Coordinating Center
Laura	Hennessey	RN	Harborview Medical Center	Los Angeles, CA, USA	Study Staff	Clinical Coordinating Center
Miriam	Hernandez		PAB	Seattle, WA, USA	Advisor	Patient Advisory Board
Karen F.	Horvath	RN, MPA	Vanderbilt University Medical Center	Nashville, TN, USA	Co-Investigator	Clinical Coordinating Center
Erin C.	Howell	MD	Harbor- University of California Los Angeles Medical Center	Los Angeles, CA, USA	Co-Investigator	Clinical Coordinating Center
Cindy	Hsu	MD, PHD	University of Michigan Medical Center	Ann Arbor, MI, USA	Co-Investigator	Clinical Coordinating Center
Jeffrey	Johnson	MD	Henry Ford Health Hospital	Detroit, MI, USA	Co-Investigator	Clinical Coordinating Center
Billie	Johnsson	MS	Weill Cornell Medical Center	New York, NY, USA	Co-Investigator	Clinical Coordinating Center
Dennis	Kim	MD	Harbor- University of California Los Angeles Medical Center	Los Angeles, CA, USA	Co-Investigator	Clinical Coordinating Center
Daniel	Kim	MD	University of Washington	Seattle, WA, USA	Co-Investigator	Clinical Coordinating Center
Tien C.	Ko	MD	University of Texas Lyndon B. Johnson General Hospital	Houston, TX, USA	Co-Investigator	Clinical Coordinating Center
Danielle C.	Lavallee	PhD	University of Washington	Seattle, WA, USA	Clinical Reviewer	Data Safety and Monitoring Board

Supplemental Online Content: Nonauthor Collaborators
*First name, last name, and suffix (if applicable) are required and will appear in PubMed.

***First Name and Middle Initial(s)**	***Last Name**	Academic Degrees	Institution	Location (city, state/province, country)	Role or Contribution, eg, chair, principal investigator	Group (if more than 1 Group listed in the byline) and/or Subgroup (eg, Steering Committee)
Debbie	Lew		University of Texas Lyndon B. Johnson General Hospital	Houston, TX, USA	Study Staff	Clinical Coordinating Center
Joseph	Mack	MD	Maine Medical Center	Portland, ME, USA	Co-Investigator	Clinical Coordinating Center
David	MacKenzie	MD	Maine Medical Center	Portland, ME, USA	Co-Investigator	Clinical Coordinating Center
Jason	Maggi	MD	Tisch Hospital NYU Langone Medical Center	New York, NY, USA	Co-Investigator	Clinical Coordinating Center
Stephanie	Marquez		University of Texas Lyndon B. Johnson General Hospital	Houston, TX, USA	Co-Investigator	Clinical Coordinating Center
Ryan	Martinez	MD	Swedish Medical Center	Seattle, WA, USA	Co-Investigator	Clinical Coordinating Center
Karen	McGrane	MD	Madigan Army Medical Center	Joint Base Lewis-McChord, WA, USA	Co-Investigator	Clinical Coordinating Center
Marcovalerio	Melis	MD	Tisch Hospital NYU Langone Medical Center	New York, NY, USA	Co-Investigator	Clinical Coordinating Center
Karen	Miller	MD	University of Washington	Seattle, WA, USA	Study Staff	Clinical Coordinating Center
Debbie	Mireles	NP	Olive View- University of California Los Angeles Medical Center	Los Angeles, CA, USA	Study Staff	Clinical Coordinating Center
Gregory J.	Moran	MD	Olive View- University of California Los Angeles Medical Center	Los Angeles, CA, USA	Co-Investigator	Clinical Coordinating Center
Dayna	Morgan	MSNBC	Swedish Medical Center	Seattle, WA, USA	Study Staff	Clinical Coordinating Center
Arden	Morris	MD, MPH	Standford University	Palo Alto, CA, USA	Clinical Reviewer	Data Safety and Monitoring Board
Kelly M.	Moser		Vanderbilt University Medical Center	Nashville, TN, USA	Study Staff	Clinical Coordinating Center
Lauren	Mount	MD	Weill Cornell Medical Center	New York, NY, USA	Co-Investigator	Clinical Coordinating Center
Kathleen	O'Connor	EdD		Seattle, WA, USA	Clinical Reviewer	Data Safety and Monitoring Board
Stephen R.	Odom	MD	Beth Israel Deaconess Medical Center	Boston, MA, USA	Co-Investigator	Clinical Coordinating Center
Oscar	Olavarria	MD	University of Texas Lyndon B. Johnson General Hospital	Houston, TX, USA	Co-Investigator	Clinical Coordinating Center
Norman	Olbrich		University of Michigan Medical Center	Ann Arbor, MI, USA	Study Staff	Clinical Coordinating Center
Scott	Osborn	MD	Virginia Mason Medical Center	Seattle, WA, USA	Co-Investigator	Clinical Coordinating Center
Olga	Owens	N-PC	DSMB	Las Vegas, NV	Clinical Reviewer	Data Safety and Monitoring Board
Pauline	Park	MD	University of Michigan Medical Center	Ann Arbor, MI, USA	Co-Investigator	Clinical Coordinating Center
Zoe	Parr	MD	University of Washington	Seattle, WA, USA	Co-Investigator	Clinical Coordinating Center
Charles S.	Parsons	MD	Beth Israel Deaconess Medical Center	Boston, MA, USA	Co-Investigator	Clinical Coordinating Center
Kavitha	Pathmarajah	MPH	Olive View- University of California Los Angeles Medical Center	Los Angeles, CA, USA	Study Staff	Clinical Coordinating Center
Deepti	Patki	MS	University of Mississippi Medical Center	Jackson, MS, USA	Study Staff	Clinical Coordinating Center
Joe H.	Patton	MD	Henry Ford Health Hospital	Detroit, MI, USA	Co-Investigator	Clinical Coordinating Center
Rebekah K.	Peacock	RN	University of Mississippi Medical Center	Jackson, MS, USA	Study Staff	Clinical Coordinating Center
Kristyn	Pierce	MS	Tisch Hospital NYU Langone Medical Center	New York, NY, USA	Co-Investigator	Clinical Coordinating Center
Kelsey	Pullar	MPH	Harborview Medical Center	Seattle, WA, USA	Study Staff	Clinical Coordinating Center
Brant	Putnam	MD	Harbor- University of California Los Angeles Medical Center	Los Angeles, CA, USA	Co-Investigator	Clinical Coordinating Center
Amy	Rushing	MD	The Ohio State University Wexner Medical Center	Columbus, OH, USA	Co-Investigator	Clinical Coordinating Center
Amber	Sabbatini	MD, MPH	University of Washington	Seattle, WA, USA	Co-Investigator	Clinical Coordinating Center
Darin	Saltzman	MD, PhD	Olive View- University of California Los Angeles Medical Center	Los Angeles, CA, USA	Co-Investigator	Clinical Coordinating Center
Matthew	Salzberg	MD	University of Mississippi Medical Center	Jackson, MS, USA	Co-Investigator	Clinical Coordinating Center
Shaina	Schaetzel	MD	Providence Regional Medical Center Everett	Everett, WA, USA	Co-Investigator	Clinical Coordinating Center
Paul J	Schmidt	MD	Olive View- University of California Los Angeles Medical Center	Los Angeles, CA, USA	Co-Investigator	Clinical Coordinating Center
Paresh	Shah	MD	Tisch Hospital NYU Langone Medical Center	New York, NY, USA	Co-Investigator	Clinical Coordinating Center
Nathan I.	Shapiro	MD, MPH	Beth Israel Deaconess Medical Center	Boston, MA, USA	Co-Investigator	Clinical Coordinating Center
Prashant	Sinha	MD	Tisch Hospital NYU Langone Medical Center	New York, NY, USA	Co-Investigator	Clinical Coordinating Center
Dionne	Skeete	MD	University of Iowa Healthcare	Iowa City, IA, USA	Co-Investigator	Clinical Coordinating Center
Elliott	Skopin	BS	PAB	Seattle, WA, USA	Advisor	Patient Advisory Board
Vance	Sohn	MD	Madigan Army Medical Center	Joint Base Lewis-McChord, WA, USA	Co-Investigator	Clinical Coordinating Center

Supplemental Online Content: Nonauthor Collaborators
*First name, last name, and suffix (if applicable) are required and will appear in PubMed.

***First Name and Middle Initial(s)**	***Last Name**	Academic Degrees	Institution	Location (city, state/province, country)	Role or Contribution, eg, chair, principal investigator	Group (if more than 1 Group listed in the byline) and/or Subgroup (eg, Steering Committee)
Lara H.	Spence	MD	Harbor- University of California Los Angeles Medical Center	Los Angeles, CA, USA	Co-Investigator	Clinical Coordinating Center
Steven	Steinberg	MD	The Ohio State University Wexner Medical Center	Columbus, OH, USA	Co-Investigator	Clinical Coordinating Center
Aleksandr	Tichter	MD	Columbia University Medical Center	New York, NY, USA	Co-Investigator	Clinical Coordinating Center
John	Tschirhart	MD	Swedish Medical Center	Seattle, WA, USA	Co-Investigator	Clinical Coordinating Center
Brandon	Tudor	MD	Providence Regional Medical Center Everett	Everett, WA, USA	Co-Investigator	Clinical Coordinating Center
Lisandra	Uribe	BA	Olive View- University of California Los Angeles Medical Center	Los Angeles, CA, USA	Study Staff	Clinical Coordinating Center
Heather	VanDusen	BS	PAB	Seattle, WA, USA	Advisor	Patient Advisory Board
Julie	Wallick	BS, BA	Swedish Medical Center	Seattle, WA, USA	Study Staff	Clinical Coordinating Center
Meridith	Weiss	MPH	PAB	Seattle, WA, USA	Advisor	Patient Advisory Board
Sean	Wells	MD	Swedish Medical Center	Seattle, WA, USA	Co-Investigator	Clinical Coordinating Center
Abigail	Wiebusch	MD	Virginia Mason Medical Center	Seattle, WA, USA	Co-Investigator	Clinical Coordinating Center
Estell J.	Williams	MD	University of Washington	Seattle, WA, USA	Co-Investigator	Clinical Coordinating Center
Robert J.	Winchell	MD	Weill Cornell Medical Center	New York, NY, USA	Co-Investigator	Clinical Coordinating Center
Jon	Wisler	MD	Ohio State University	Columbus, OH, USA	Co-Investigator	Clinical Coordinating Center
Bruce	Wolfe	MD	Maine Medical Center	Portland, ME, USA	Clinical Reviewer	Data Safety and Monitoring Board
Erika	Wolff	PhD	Harborview Medical Center	Seattle, WA, USA	Co-Investigator	Executive Committee
Donald M.	Yealy	MD	University of Pittsburgh Medical Center	Jackson, MS, USA	Clinical Reviewer	Data Safety and Monitoring Board
Julianna	Yu	MD	Virginia Mason Medical Center	Seattle, WA, USA	Co-Investigator	Clinical Coordinating Center
