# Supplementary material for: Association of Patient Belief About Success of Antibiotics for Appendicitis and Outcomes: A Secondary Analysis of the CODA Randomized Clinical Trial
Source: JAMA Surg. 2022 Oct 5;157(12):1080–7. doi: 10.1001/jamasurg.2022.4765 (PMC9535504; doi:10.1001/jamasurg.2022.4765)
Supplement: Supplement 5. — Data Sharing Statement [file jamasurg-e224765-s005.pdf]

Data Sharing Statement

Zhang. Association of Patient Belief About Success of Antibiotics for Appendicitis and Outcomes. *JAMA Surg*. Published October 05, 2022. doi:10.1001/jamasurg.2022.4765

Data
Data available: No
